# High CD34 surface expression in BCP‐ALL predicts poor induction therapy response and is associated with altered expression of genes related to cell migration and adhesion

**DOI:** 10.1002/1878-0261.13207

**Published:** 2022-04-07

**Authors:** Signe Modvig, Rasmus Wernersson, Nina Friesgaard Øbro, Lars Rønn Olsen, Claus Christensen, Susanne Rosthøj, Matilda Degn, Gitte Wullf Jürgensen, Hans O. Madsen, Birgitte Klug Albertsen, Peder Skov Wehner, Steen Rosthøj, Henrik Lilljebjörn, Thoas Fioretos, Kjeld Schmiegelow, Hanne Vibeke Marquart

**Affiliations:** ^1^ Department of Clinical Immunology Copenhagen University Hospital Rigshospitalet Denmark; ^2^ 4321 Faculty of Medicine Institute of Clinical Medicine University of Copenhagen Denmark; ^3^ 5205 Section for Bioinformatics Department of Health Technology Technical University of Denmark Lyngby Denmark; ^4^ 5205 Intomics A/S Lyngby Denmark; ^5^ 4321 Section of Biostatistics University of Copenhagen Denmark; ^6^ Department of Pediatric and Adolescent Medicine Copenhagen University Hospital Rigshospitalet Denmark; ^7^ 11297 Department of Pediatrics and Adolescent Medicine Aarhus University Hospital Denmark; ^8^ Department of Clinical Medicine Aarhus University Denmark; ^9^ H.C. Andersen Children's Hospital Odense University Hospital Denmark; ^10^ 53141 Department of Pediatrics and Adolescent Medicine Aalborg University Hospital Denmark; ^11^ 5193 Division of Clinical Genetics Department of Laboratory Medicine Lund University Sweden

**Keywords:** acute lymphoblastic leukemia, CD34, cell migration, immunophenotype, prognosis, protein–protein interaction networks

## Abstract

Minimal residual disease (MRD) constitutes the most important prognostic factor in B‐cell precursor acute lymphoblastic leukemia (BCP‐ALL). Flow cytometry is widely used in MRD assessment, yet little is known regarding the effect of different immunophenotypic subsets on outcome. In this study of 200 BCP‐ALL patients, we found that a CD34‐positive, CD38 dim‐positive, nTdT dim‐positive immunophenotype on the leukemic blasts was associated with poor induction therapy response and predicted an MRD level at the end of induction therapy (EOI) of ≥ 0.001. CD34 expression was strongly and positively associated with EOI MRD, whereas CD34‐negative patients had a low relapse risk. Further, CD34 expression increased from diagnosis to relapse. CD34 is a stemness‐associated cell‐surface molecule, possibly involved in cell adhesion/migration or survival. Accordingly, genes associated with stemness were overrepresented among the most upregulated genes in CD34‐positive leukemias, and protein–protein interaction networks showed an overrepresentation of genes associated with cell migration, cell adhesion, and negative regulation of apoptosis. The present work is the first to demonstrate a CD34‐negative immunophenotype as a good prognostic factor in ALL, whereas high CD34 expression is associated with poor therapy response and an altered gene expression profile reminiscent of migrating cancer stem‐like cells.

## Graphical abstract

This study reports that a leukemic cell immunophenotype with high CD34 surface expression predicts poor induction therapy response in B‐cell precursor acute lymphoblastic leukemia. Moreover, blast CD34 expression increases from diagnosis to relapse. Protein–protein interaction network analysis and gene set enrichment analysis show increased expression of genes associated with stemness, cell adhesion, cell migration, and cell survival in CD34‐positive cases.

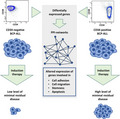

**Abbreviations**
AIC: Akaike information criterion
ALL: acute lymphoblastic leukemia
AML: acute myeloid leukemia
AUC: area under the curve
BCP: B‐cell precursor
BM: bone marrow
CG: cytogenetic group
CIR: cumulative incidence of relapse
CNS: central nervous system
EFS: event‐free survival
EOI: end of induction therapy
FCM: flow cytometry
FDR: false discovery rate
GO: gene ontology
GSEA: gene set enrichment analysis
HSC: hematopoietic stem cells
Ig: immunoglobulin
LAIP: leukemia‐associated immunophenotype
LSC: leukemic stem cells
MFI: median fluorescence intensity
MRD: minimal residual disease
NOPHO: Nordic Society of Pediatric Hematology and Oncology
NPV: negative predictive value
PAM: prediction analysis for microarrays
PB: positive bright expression
PCA: principal component analysis
PCR: polymerase chain reaction
PD: positive dim expression
PN: positive normal expression
PPI: protein–protein interaction
ROC: receiver operating characteristic
TCR: T‐cell receptor
WBC: white blood cell count

## 1 Introduction

The level of minimal residual disease (MRD) at the end of induction (EOI) therapy is the single most important prognostic factor for clinical outcome in B‐cell precursor (BCP)‐ALL [1, 2], associating closely with the risk of relapse [3]. The leukemia‐associated immunophenotype (LAIP) forms the basis for BCP‐ALL diagnosis and MRD monitoring by flow cytometry (FCM) [3]. Specific immunophenotypic markers have been associated with distinct cytogenetic groups (CG) [4, 5, 6, 7, 8] and have been investigated in relation to prognosis, with varying findings [6, 9, 10, 11, 12]. However, immunophenotypic heterogeneity is common in BCP‐ALL [13], compromising accurate classification based on LAIP, and so this heterogeneity should be accounted for in the LAIP characterization to properly assess whether a relationship between LAIP and prognosis exists.

Apart from its role in diagnosis and MRD monitoring, the LAIP can be linked to properties providing the cells with survival advantages in a chemotherapy setting, thus indicating a prognostic potential for the LAIP. This has been shown for surface markers such as integrin alpha 4 (CD49d) and alpha 6 (CD49f) in relation to CNS homing [14], bone marrow niche adherence [15], chemotherapy resistance through stromal cell signaling [16, 17, 18, 19], and persistent MRD with poor clinical outcome [20, 21]. In AML, CD34 expression has been associated with poor clinical outcome [22, 23, 24], and CD38‐negative CD34‐positive leukemic cells demonstrate increased leukemia‐initiating capacity and show stem‐like features, a quiescent phenotype and increased expression of adhesion‐related molecules such as CD44, CXCR4, and integrins, as well as of the growth guidance receptor ROBO4 [25, 26]. In ALL, a recent report showed that co‐culture of leukemic cells with BM mesenchymal stromal cells led to upregulation of CD34 and downregulation of CD38 along with increased adherence, dormancy, and therapy resistance [27]. At present, these findings have not been corroborated in a clinical setting of ALL and no data firmly associate immunophenotypes including CD34 with clinical outcome.

In this study, we examined the LAIP of BCP‐ALL and investigated its association with therapy response and relapse taking the immunophenotypic heterogeneity into account. We show that high CD34 expression is associated with poor therapy response as measured by MRD at EOI and that clinical cases of CD34‐positive ALL express genes associated with stemness, migration, adhesion, and survival.

## 2 Materials and methods

### 2.1 Subjects

We retrospectively evaluated flow cytometry data from time of diagnosis in 200 patients (172 children < 18 years and 28 adults 18–45 years) diagnosed with BCP‐ALL between October 2009 and June 2015, treated, and monitored in Denmark by the standardized Nordic Society of Pediatric Hematology and Oncology (NOPHO) ALL2008 protocol [28]. Age, gender, white blood cell count (WBC), cytogenetic aberrations/karyotyping, and treatment stratification were registered. Further, levels of MRD by FCM and PCR at end of induction therapy (day 29) as well as follow‐up information on relapse, death, and secondary malignancy were registered. All analyses were undertaken with the understanding and written consent of each subject including consent of a legal guardian for minors. The study methodologies conformed to the standards set by the Declaration of Helsinki, and the Capital Regional Ethics Committee approved the study (H‐2‐2010‐002).

### 2.2 Sample processing, flow cytometric analysis at diagnosis, and MRD analysis

Bone marrow (BM) was sampled at time of diagnosis and day 29 according to the ALL2008 protocol guidelines, as previously described [3]. BM samples were subjected to 6‐color FCM analysis, ensuring a high degree of standardization over the inclusion period by normalization to Rainbow beads. At least 50 000 events were acquired at diagnosis although if material was available, 100 000 events per marker‐combination were analyzed to ensure optimal identification of subpopulations.

At MRD timepoints, the first aspirate was used for FCM‐ and PCR‐MRD to avoid hemodilution and subsequent differences in assessment of blast concentration, and the bone marrow material was split equally for FCM‐ and PCR‐MRD. MRD was measured by flow cytometry using protocol‐defined six‐color panels for identification and monitoring of the LAIP according to the NOPHO ALL2008 guidelines [3, 28]. At least 300 000 events, but preferably 1 million events, per antibody combination were analyzed when sufficient material was available, corresponding to a sensitivity of 1 × 10^−5^ (sensitivity calculated as 10/live singlets, corresponding to the identification of ≥ 10 clustered leukemic events among all live singlets analyzed, as defined by the ALL2008 protocol [3]). For PCR‐based confirmation of the association between LAIP and EOI MRD, MRD was measured in 65/200 patients by real‐time quantitative PCR using clone‐specific TCR/Ig primers according to the EuroMRD guidelines [29, 30].

### 2.3 LAIP scoring

The expression of intracellular and surface B‐lineage markers (CD19, CD20, CD22, nTdT, cyCD79a, and cyCD22), nonlineage (CD45, CD34, CD38, CD10, and nTdT), and cross‐lineage expressed markers (CD123, CD66c, CD133, CD13, CD33, and CD15) was scored as negative (neg, −) or positive [dim (PD, +), normal (PN, ++) or bright (PB, +++)] using standardized reference intervals based on marker fluorescence intensity (FI) levels on normal bone marrow lymphocyte subsets. Five non‐ALL bone marrow samples, evenly distributed over the inclusion period and with unaffected B‐lymphopoiesis, were used for defining reference intervals to ensure the robustness of reference levels over the full inclusion period, while allowing for comparison with relevant normal counterparts, which are not always present in leukemic bone marrow samples at time of diagnosis (Fig. S1 and Table S1). For a complete classification of the leukemia, additional T‐lineage (including cytCD3, CD2, and CD7) and myeloid lineage (including CD117 and cytoplasmic MPO) antigens were analyzed. Bimodal expression was defined as separate populations with distinct peaks in contour plot (resolution 68 and percentage 10) and histograms comprising more than 1% of the blast population. The 1% limit was chosen to accommodate the fact that small subsets, comprising < 5% at diagnosis, can in some cases comprise the majority of the residual disease at end of induction [3]. Broad expression was defined as a population with only one peak extending minimum 1.5 score (for B‐ and nonlineage markers, Table S1) or decade (for cross‐lineage markers), using the 10% contour line as population boundary.

Bimodally expressed antigens were given a score for each subpopulation and an overall score according to that of the dominant subpopulation. For analyses of immunophenotype and outcome, this overall score was applied in patients with bimodal marker expression, thus accounting for the immunophenotypic heterogeneity. Median fluorescence intensities (MFI) and relative distributions of subpopulations were registered for CD34 and CD38. All FCM files were reviewed by the same physician to eliminate interobserver bias. All samples were run on a FACS Canto and analyzed in diva 6.0 software (BD, Franklin Lakes, NJ, USA).

### 2.4 Gene expression analysis, protein–protein interaction analysis, and identification of Ph‐like cases

Gene expression analysis was performed on bone marrow in all patients with available material and consent at time of diagnosis [*n* = 160 (80%), human gene 1.0 ST array; Affymetrix, Thermo Fisher Scientific, Waltham, MA, USA]. Probe intensities were read using the Oligo package [31] and normalized using Robust Multiarray Average; batch effects were corrected using combat [32], and differentially expressed genes were identified by two group comparisons using an FDR‐corrected *P*‐value (*q*‐value, Benjamini–Hochberg [33]) of 0.05 as cutoff level using limma [34]. These genes were used for principal component analysis (PCA) and overrepresentation analyses. Protein–protein interaction (PPI) network analysis was performed using the high‐confidence interactions (confidence threshold = 0.119) of the April 2019 build of inBio Map human interactome resource [35]. In order to identify significantly regulated subnetworks, we used the in‐house algorithm ‘SystemSignificance’ which has previously been detailed [36]. Briefly, the algorithm works by iteratively assessing the 1st‐order network around each human protein, by (a) mapping in the corresponding *P*‐values for each member of the network from the gene expression dataset, (b) integrating the *P*‐values using Edgingtons method, and (c) shuffling and resampling the data values in the dataset 10^7^ times to evaluate the chance at obtaining an integrated *P*‐value equal or more extreme in a network of this size by coincidence. In this study, we selected the networks (*n* = 10) where < 1000 of the 10^7^ permutations gave rise to equal or better *P*‐values. This corresponds to a false discovery rate of ~ 15% estimated on permutation of the input data. The resultant networks were collected in cytoscape for initial inspection and data‐sharing purposes and further analyzed and visualized using the inBio Discover online tool (https://inbio‐discover.com/). Biological function of each network was assessed using Gene Ontology (GO) enrichment analysis either using the online Panther tool (http://geneontology.org/), or using the enrichment analysis module of inBio Discover tool. Gene set enrichment analysis was performed using the moderated *t*‐statistic for ranking differential expression of genes between CD34‐negative and CD34‐positive leukemias using the r package fgsea for (a) identification of top enriched pathways using Reactome, (b) test of HSC signature enrichment, and (c) validation of GO biological processes identified in the PPI networks.

Cases with Ph‐like gene expression were identified by three strategies, based on 203 probes on the HuGene 1.0 ST array. These probes represented 188 of the 195 genes detected by the 257 probes on the U133 Plus 2.0 platform from the original PAM classifier for identifying Ph‐like cases [37], Hence, all genes from the original PAM classifier, where a corresponding probe could be identified on the HuGene 1.0 ST array, were included. First, we performed a mock PAM classification by calculating the total sum of all gene expression values multiplied with the PAM factor described by Roberts et al. [37] and selected the 5% of samples with the highest score. For genes with multiple PAM factors (i.e., genes detected by multiple probes), the mean PAM factor was used. Secondly, we performed hierarchical clustering based on all 203 probes and selected all cases in the cluster with the most compatible gene expression profile according to the PAM factor. Lastly, we filtered out the 25 most significant genes out of these 170 when comparing Ph‐like and other cases in an independent dataset [38] and performed hierarchical clustering based on those genes. The cases in the cluster with the most compatible gene expression for a Ph‐like profile were selected. All cases selected by at least two of these three strategies were considered having Ph‐like gene expression. In total, 10 Ph‐like cases were identified with this strategy. Of those, nine were selected by all three methods and one was selected by two of the three methods.

### 2.5 Statistical analysis

End of induction therapy MRD by flow cytometry was used as primary outcome. MRD results below the individual lower limits of detection were set to 10^−5^. Immunophenotypic markers and clinical characteristics were analyzed as explanatory variables using linear regression, including correction for multiple testing by the Bonferroni method. The best combination of variables was examined in a multiple regression analysis using forward and backward selection as well as least absolute shrinkage and selection operator (LASSO) based on the Akaike Information Criterion (AIC). Variables selected by either of the three methods and with *P*‐values below 0.05 were included in the final model. Receiver operating characteristics (ROC) curves were generated using MRD ≥ 0.001 as outcome and the area under the curve (AUC) was used to estimate predictive value of variables. For ROC analyses and for validation of four‐category scoring in multiple regression analysis, markers were classified as binary (neg/dim vs pos/bright for all markers, except for CD15 and CD133, which were neg vs dim/pos/bright) due to small numbers in some subgroups). For paired analyses, Wilcoxon signed‐rank test was used. Spearman's rank correlation was used to test leukemic subpopulation distributions in relation to EOI MRD. The Kaplan–Meier method was used to determine 5‐year event‐free survival (EFS_5y_), and the Aalen–Johansen estimator was used to determine 5‐year cumulative incidence of relapse (CIR_5y_) treating death and secondary malignancy as competing risks, and Wald test was used to test for differences in CIR. Cause‐specific Cox regression analyses were used to study the association between immunophenotypic markers and relapse with censoring by death or secondary malignancy. Linearity of quantitative variables and the proportional hazards assumption were assessed using Martingale residuals [39]. Analyses were performed in sas 9.4 (SAS Institute Inc, Cary, NC, USA) and r 3.6.0.

## 3 Results

### 3.1 The immunophenotype predicts response to induction therapy

We examined the LAIP and mapped immunophenotypic heterogeneity at time of diagnosis in 200 patients with BCP‐ALL (demographic and clinical characteristics in Table 1). Of the 200, 108 (54%) had > 1 immunophenotypic subpopulation at diagnosis (Fig. S2A). An additional 77 patients (39%) had broad expression of one or more markers. The most commonly heterogeneously expressed markers were CD34 (50%), CD20 (43%), CD66c (34%), CD45 (21%), CD10 (19%), and nTdT (15%) (Fig. S2B,C).

**Table 1:** Patient data. For continuous variables median (IQR, range) is given, for categorical variables number (%) is given. LOD, limit of detection.

Clinical characteristics	Value
Age	5 (3–13, 1–44) years
Gender	93/105 (47/53%) male/female
WBC	9 (4–28, 0.7–388) x 10^9^·L^−1^
High hyperdiploid	64 (32%)
Hypodiploid	6 (3%)
t(12;21)	35 (18%)
t(1;19)	5 (3%)
iAMP21	7 (4%)
dic(9;20)	2 (1%)
*KMT2A*‐rearranged	4 (2%)
No cytogenetic aberration	77 (39%)
EOI risk stratification: SR/IR/HR/HR+HSCT	84/89/13/12 (42/45/7/6%)
EOI FCM‐MRD	1.3 × 10^−3^ (7.4 × 10^−5^–4.7 × 10^−3^, < LOD‐6.7 × 10^−1^)
Follow‐up time	73 (57–92, 2–117) months
Relapse	18 (9.0%, CI 4.9–13.0%^a^)
Nonrelapse mortality	9 (4.6%, CI 1.7–7.5%^a^)

^a^ 5‐year cumulative incidence of relapse/cumulative incidence of nonrelapse mortality.

**Fig. 1:**
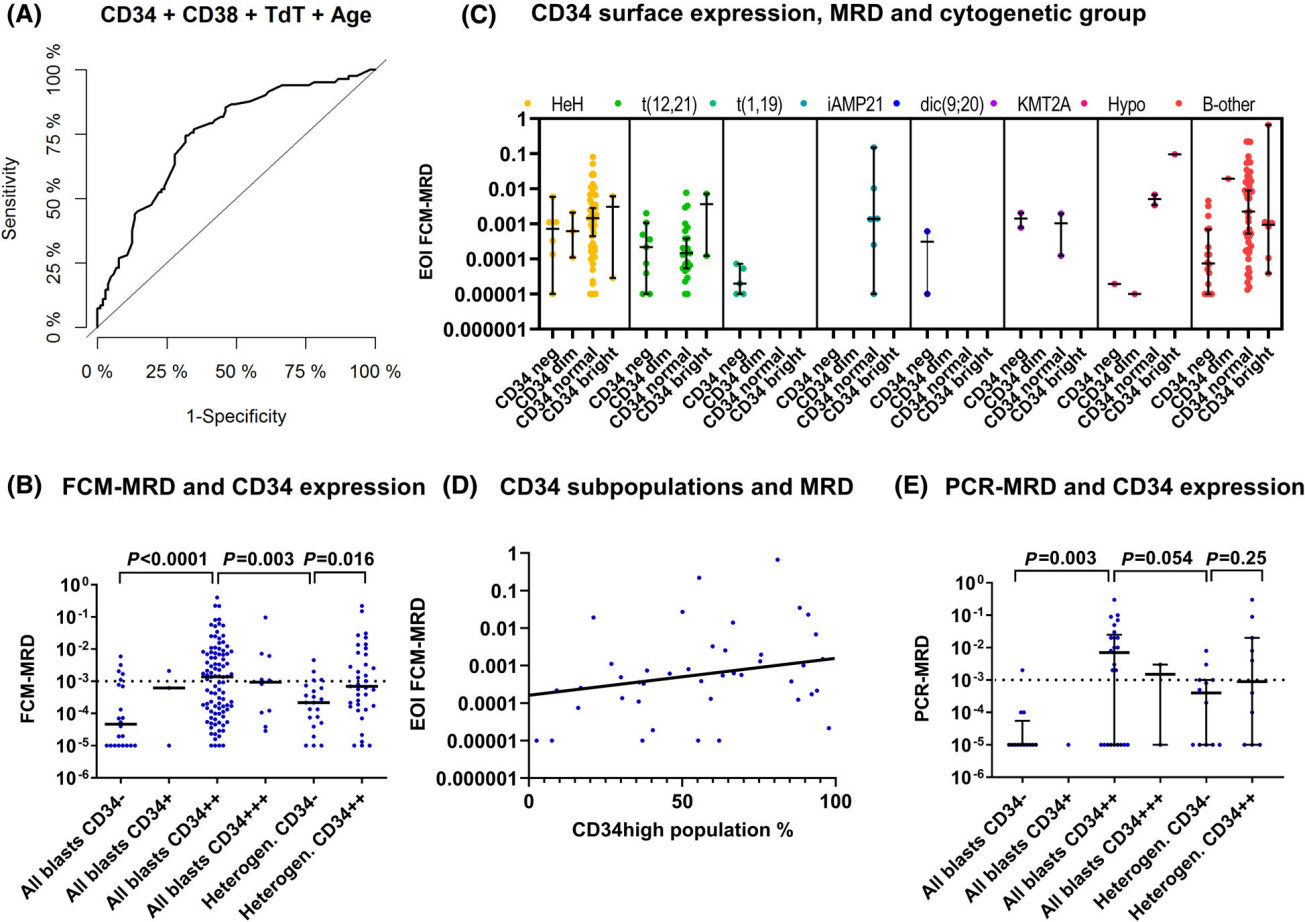
CD34 surface expression and minimal residual disease. (A) Receiver operating characteristic curve with end of induction MRD level > 10^−3^ as outcome and with CD34, CD38, nTdT, and patient age at time of diagnosis as predictors showing an AUC of 0.75 (CI 0.68–0.82, *n* = 185). (B) CD34 surface expression and end of induction (day 29) MRD level measured by flow cytometry (*n* = 191). Grouping patients with heterogeneous CD34 expression by the score of the dominant subpopulation yields equal distributions among uni‐ and bimodal cases with regard to EOI MRD. Line represents median level. Horizontal dashed line refers to the end of induction stratification level in the NOPHO ALL2008 protocol. *P*‐values from Mann–Whitney *U* test. (C) End of induction MRD level and CD34 surface expression, by cytogenetic subgroup (*n* = 191). The CD34 expression is illustrated as a combined score, where cases with bimodal and unimodal expression were pooled according to the score of the dominant subpopulation in cases with bimodal expression and the score of the whole population in cases with unimodal expression. (D) Association between end of induction MRD level and the relative distribution of CD34 subpopulations (*n* = 42). In one case, both populations were CD34‐positive and so the CD34‐positive population was set to 100%. (E) CD34 surface expression and end of induction MRD measured by PCR (*n* = 65). Lines represent median and IQR. Horizontal dashed line refers to the end of induction stratification level in the NOPHO ALL2008 protocol. *P*‐values from Mann–Whitney *U* test. EOI, end of induction; FCM, flow cytometry; IQR, interquartile range; LOD, limit of detection; MRD, minimal residual disease; NOPHO, Nordic Society of Pediatric Hematology and Oncology.

Immunophenotypic markers were scored as negative (neg, −) or positive [dim (PD, +), normal (PN, ++) or bright (PB, +++)] and heterogeneity in expression for each marker was accounted for, as described in the methods section. The LAIP was then examined in relation to induction therapy response, where the expression of three immunophenotypic markers at time of diagnosis was significantly associated with the level of MRD at EOI (CD34, CD38, and CD66c; univariate linear regression, Table 2), but only CD34 and CD38 were significant after correction for multiple testing. Notably, the same result was obtained when dividing the markers into only two categories (neg/dim vs pos/bright, data not shown). To identify a potential high‐risk immunophenotype, we investigated which combination of immunophenotypic markers and known risk factors (age, WBC, and cytogenetic subtype) comprised the best explanatory model for induction therapy response (multiple regression, Table 2). A CD34‐positive, CD38 dim‐positive, and nTdT dim‐positive phenotype was found to be associated with a poor induction therapy response, and this immunophenotype predicted an EOI MRD level above the protocol SR/IR stratification cutoff level of 0.001 (AUC 0.70, CI 0.63–0.77). In combination with age, the predictive value increased to 0.75 (CI 0.68–0.82) (Fig. 1A), while WBC or cytogenetic subgroup did not add predictive value. Patients with a CD34‐positive, CD38 dim‐positive, and nTdT dim‐positive phenotype had a 41‐fold (CI 4.9–341) increased MRD level compared with other patients (*P* = 0.0007), while patients with a CD34‐positive CD38 dim‐positive phenotype, regardless of nTdT expression, had a 4.3‐fold increased MRD level compared with other patients (*P* = 0.0001).

**Table 2:** Immunophenotypic marker expression and minimal residual disease. Markers were tested in univariate and multiple regression analysis with log‐transformed end of induction (day 29) minimal residual disease level as outcome. Markers were analyzed as categorical variables based on marker expression levels, using a combined score with four levels (negative/PD/PN/PB), where cases with bimodal and unimodal expression were pooled according to the score of the dominant subpopulation in cases with bimodal expression and the score of the whole population in cases with unimodal expression. The effects are estimates of the ratio between the MRD levels for the given marker expression level compared with the reference group. CD34 reference group: CD34 negative. CD38 reference group: CD38PN. nTdT reference group: nTdTPN. Age was treated as a quantitative variable, where the effect on MRD corresponds to a 10‐year increase in age. PB, positive bright; PD, positive dim; PN, positive normal; WBC, white blood cell count (peripheral blood).

Marker	Univariate	Multiple	Effect
CD34	< 0.0001	0.0007	6.47 (CI 2.56–16.32) for CD34PN
CD38	0.0022	0.0297	2.57 (CI 1.21–5.46) for CD38PD
CD10	0.0903		
CD20	0.89		
CD19	0.94		
CD45	0.29		
CD22	0.52		
nTdT	0.0771	0.0085	4.58 (CI 1.43–14.67) for nTdTPD
CyCD22	0.88		
CyCD79alfa	0.27		
CD133	0.0677		
CD13	0.60		
CD66c	0.0252		
CD33	0.48		
CD15	0.41		
Age	0.0292	0.0607	1.28 (CI 0.99–1.66)
WBC at diagnosis	0.78		
Cytogenetic group	0.0038		

### 3.2 CD34 surface expression associates with EOI MRD independently of other risk factors

CD34 was the marker most closely associated with induction therapy response with a 6.47‐fold increase in MRD for CD34PN compared with CD34 negative (*P* = 0.0007, multiple regression, Table 2), and a CD34 negative/predominantly negative immunophenotype predicted a low EOI MRD level [negative predictive value (NPV) = 0.79 for MRD < 0.001, NPV = 1 for MRD < 0.01]. When accounting for heterogeneity, the uni‐ and bimodal cases showed equal distributions with regard to the EOI MRD response (Fig. 1B). The association between CD34 expression and induction therapy response was independent of other known risk factors, such as cytogenetic subgroup, WBC, and age (*P* = 0.0002 for CD34, multiple regression). Some cytogenetic subgroups seemed to have a distinct CD34 expression profile but were too small to evaluate separately (Fig. 1C and Table S2A), while CD34 expression varied within the high hyperdiploid subgroup and within patients with no identified cytogenetic aberrations (B‐other), where it also associated with EOI MRD level (CD34 negative vs CD34PN: *P* = 0.0001, Fig. 1C). There was no significant association between CD34 expression and WBC (*P* = 0.11) or age (*P* = 0.19, Kruskal–Wallis test).

For leukemias with bimodal CD34 expression, we found a positive correlation between the percentage of leukemic cells in the CD34‐positive subpopulation and the EOI MRD level (*r* = 0.34, *P* = 0.0254, *n* = 42, Spearman's rank correlation, Fig. 1D). Additionally, increased CD34 MFI was associated with increased MRD levels: a 10‐fold increase in CD34 expression corresponded to a 2.4‐fold increase in EOI MRD (*P* = 0.0005, linear regression, *n* = 152). Thus, both the number of CD34‐positive cells within the leukemia and the overall CD34 MFI level seemed to be associated with the induction therapy response.

In the ALL2008 protocol, BCP‐ALL patients were primarily stratified by FCM‐MRD [3]. Hence, the association between CD34 and therapy response could potentially be biased, if a CD34‐positive immunophenotype was more informative, thus underestimating MRD in CD34‐negative leukemias. We, therefore, measured the MRD level by PCR in CD34‐negative/predominantly negative cases with available DNA using a sensitive PCR marker (*n* = 26). A corresponding subset of CD34‐positive cases with high and low MRD levels, respectively, were also analyzed (*n* = 39, 22 with MRD > 10^−3^). This confirmed the association between CD34 and EOI MRD level by PCR (20.5‐fold increase (CI 3.7–379.7) in EOI MRD for CD34‐positive vs CD34‐negative, *P* = 0.0058, linear regression, and Fig. 1E).

### 3.3 CD34 surface expression increases from diagnosis to relapse

**Fig. 2:**
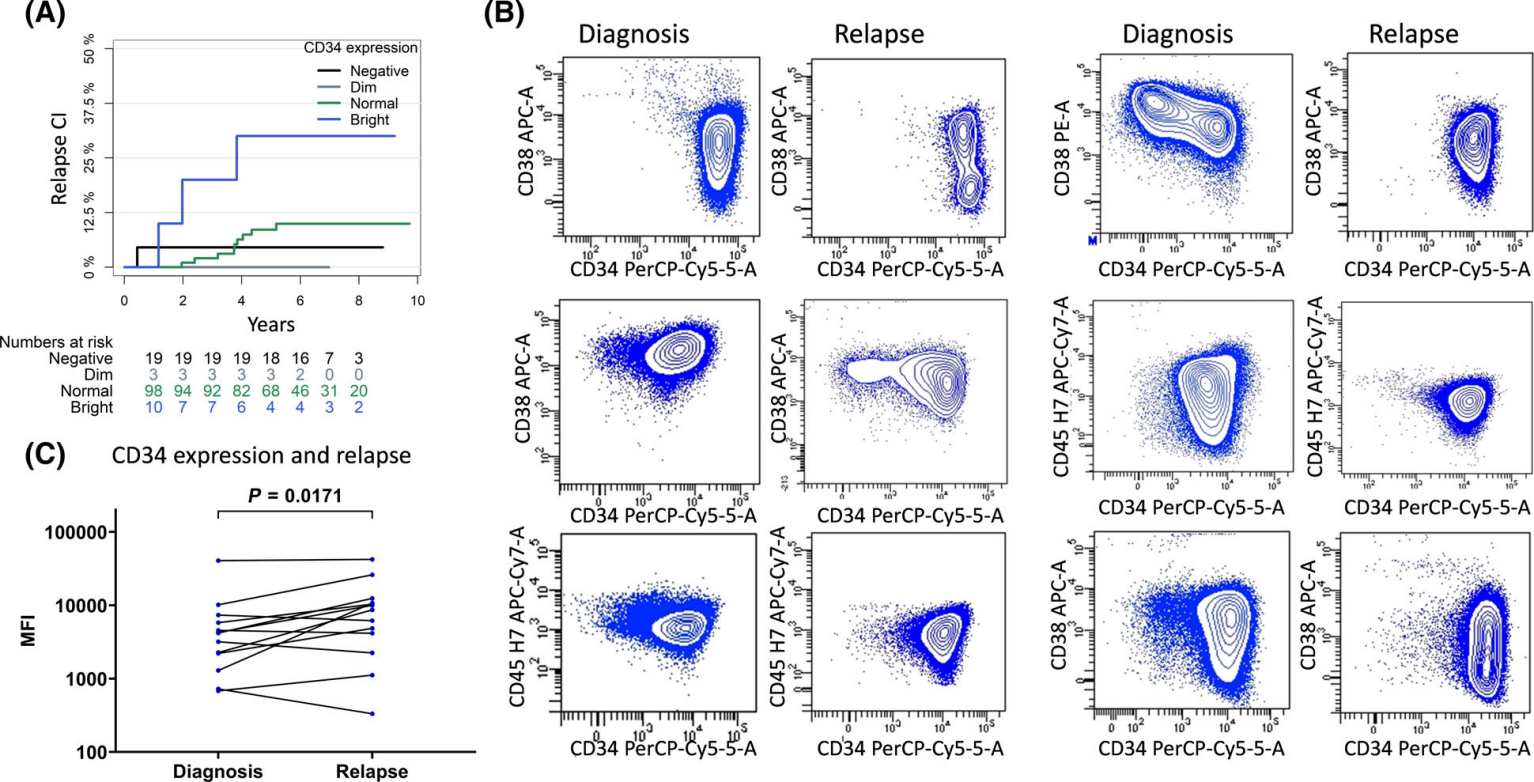
CD34 surface expression and relapse. (A) CD34 surface expression and cumulative incidence of relapse in patients with unimodal CD34 expression (*n* = 134). (B) CD34 surface expression at diagnosis and at time of relapse in the 6/13 patients with available flow cytometry at time of relapse, who displayed a CD34 bright immunophenotype at time of relapse. A simultaneous decrease in CD38 expression was observed for the 4/6 cases, where CD38 was measured. For the case in the top right corner, CD34 was conjugated with FITC at diagnosis. (C) CD34 showed an overall increase in surface expression from diagnosis to time of relapse in the 13 patients with available flow cytometry data at time of relapse (Wilcoxon signed‐rank test).

Even though EOI MRD is closely related to the risk of relapse, certain ALL subtypes show delayed clearance of MRD cells, yet a low relapse incidence [40]. Since CD34 showed the strongest association with induction therapy response of all the markers, we examined the direct association between CD34 expression and relapse. The overall 5‐year event‐free survival and CIR_5y_ of this cohort were 86.5% (CI 81.7–91.4%) and 9.0% (CI 4.9–13%). Although there was an increased CIR_5y_ with increased CD34 expression in patients with unimodally expressed CD34, this association was not significant, perhaps due to a low number of relapses (*n* = 18) in the cohort [CD34 negative: CIR_5y_ 4.6% (CI 0–13%, *n* = 22), CD34PD: CIR_5y_ 0% (CI 0–0%, *n* = 4) CD34PN: CIR_5y_ 8.6% (CI 2.9–14%, *n* = 98), and CD34PB: CIR_5y_ 30% (CI 1.6–58%, *n* = 10), Fig. 2A]. For the patients with bimodal expression, this trend was not observed [CIR_5y_ 13.6% (CI 7.3–28%, *n* = 23] for CD34 predominantly negative, CIR_5y_ 6.3% (CI 0–14.7%, *n* = 36) for CD34 predominantly positive). In accordance with this, a higher CD34 MFI did not significantly increase the risk of relapse in patients with unimodal CD34 expression (HR 1.45 for a 10‐fold increase in CD34 MFI, CI 0.58–3.62, *P* = 0.43, *n* = 110, 13 events, cause‐specific Cox regression). However, in patients with available flow cytometry data at time of relapse (*n* = 13), 46% had a CD34PB immunophenotype, compared to only 5% at diagnosis (Fig. 2B). Furthermore, there was an overall increase in CD34 expression from diagnosis to relapse among all patients (*P* = 0.0171, Fig. 2C).

### 3.4 Genes associated with stemness, migration, adhesion, and survival are abundantly expressed in CD34‐positive leukemia

Gene expression was analyzed in 160/200 cases (CG distribution shown in Table S2B). For all markers included in the flow cytometric profiling, good consistency between mRNA and surface protein expression was observed (Fig. S3), including a moderate correlation between expression of the CD34 and CD38 genes and surface expression of CD34 (*r* = 0.41, *P* < 0.0001, *n* = 134) and CD38 (*r* = 0.53, *P* < 0.0001, *n* = 134). Since CD34 was the marker with the strongest association to therapy response, we compared the gene expression profiles of CD34‐positive (normal or bright, *n* = 87) and CD34‐negative (*n* = 18) cases, resulting in 551 differentially expressed genes with a *q*‐value < 0.05. A PCA analysis separated the groups clearly (Fig. S4), suggesting that differences in CD34 surface expression indeed reflected a more generalized variation between the groups.

**Fig. 3:**
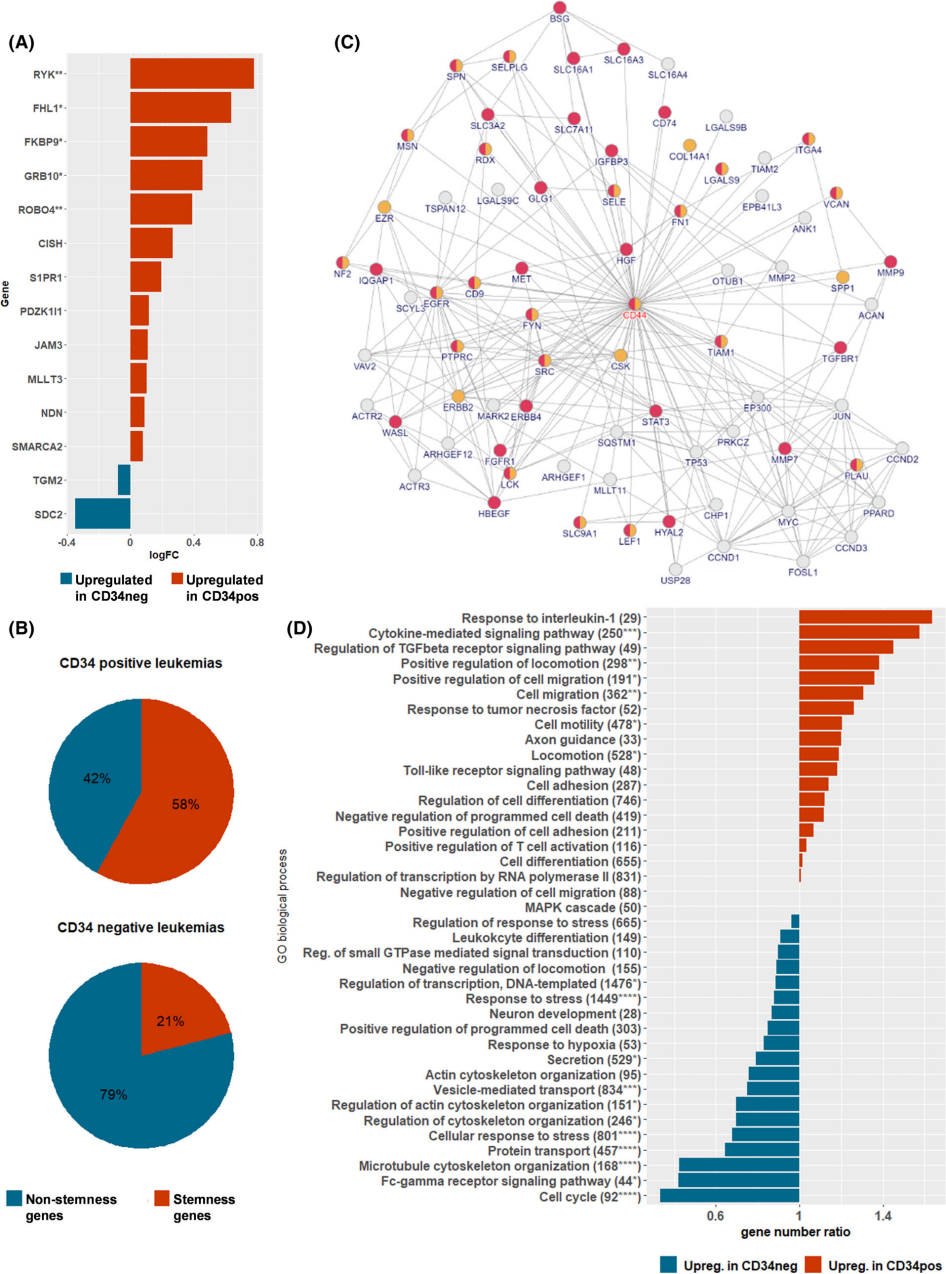
Annotations of differentially expressed genes between CD34‐positive and CD34‐negative leukemias. (A) Fourteen genes, found to be upregulated in normal, quiescent HSCs and validated by qPCR by Forsberg et al. [41] were evaluated for expression in CD34‐positive (*n* = 87) and CD34‐negative leukemias (*n* = 18). Expression of 12/14 was higher in CD34‐positive leukemias, two (*ROBO4* and *RYK*) significantly after FDR correction (Benjamini–Hochberg). *P < 0.05, **q < 0.05. (B) The top 50 differentially expressed genes between CD34‐positive and CD34‐negative leukemias were evaluated for association with stemness in the literature (Table S3). 18/26 for CD34 positive vs 8/24 for CD34 negative were found to associate with stemness, *P* = 0.0225, Fisher's exact test. (C) Network 5 with seed protein CD44. Edges represent known protein interactions, and nodes represent proteins, with gene names encoding the proteins in capital letters below. Yellow nodes mark genes involved in cell adhesion (cell adhesion GO:0007155, regulation of cell adhesion GO:0030155, positive regulation of cell adhesion GO:0045785, and negative regulation of cell adhesion GO:0007162), red nodes mark cell migration (cell migration GO:0016477, regulation of cell migration GO:0030334, positive regulation of cell migration GO:0030335, and negative regulation of cell migration GO:0030336). (D) Direction of gene expression in enriched GO biological processes from PPI networks. Processes, found to be overrepresented in at least one of the 10 PPI networks (Table S4) and comprising more than 10 genes, are included. For each GO process, the number of genes upregulated more than 0.1 log2FC in CD34‐positive vs CD34‐negative leukemias and vice versa were registered and the ratio between the two illustrated. The name of the process is followed by the number of genes in the process and a *P*‐value testing distribution of genes upregulated in CD34 positive vs CD34 negative within each GO process vs distribution in remaining genes (total: 8709 upreg. in CD34 negative, 8490 upreg. in CD34 positive) using Chi‐square test with Yates' correction (*P < 0.05, **P < 0.01, ***P < 0.001, ****P < 0.0001).

Given the existing knowledge of CD34 in cancer cells, we investigated our dataset for possible associations between CD34 expression and stemness, migration, adhesion, and survival hoping to gain insight into the causes of the generalized variation. With respect to stemness, we looked for possible enrichment of 93 genes found to associate with steady‐state, quiescent HSC in a study by Forsberg et al. [41], who demonstrated a significant overlap with six other studies. Although we found no overall significance of differential expression of these genes between the CD34‐positive and CD34‐negative leukemias comparing the gene level *P*‐values against the global *P*‐value distribution, we did see a significant enrichment of the genes in the CD34‐positive leukemias in the rank‐based gene set enrichment analysis (GSEA) (*P* = 0.0007, Fig. S5A). Furthermore, 12 of the 14 key HSC genes, chosen for validation by Forsberg et al., showed higher expression among the CD34‐positive leukemias, two of which remained significant after FDR correction [*RYK* (*q* = 0.03) and *ROBO4* (*q* = 0.011) Fig. 3A]. Accordingly, examining the top 50 differentially expressed genes in our dataset (Table S3) showed a higher proportion of genes, associated with stemness in the literature, and upregulated in the CD34‐positive leukemias (*P* = 0.0225, Fisher's exact test, Fig. 3B). Hence, CD34‐positive ALL appeared to express a number of genes, which like CD34 itself have been associated with stemness.

The examination of the top 50 genes in relation to gene ontology (GO) biological process annotations revealed that genes involved in signaling or adhesion/migration comprised 50% of the genes upregulated in CD34‐positive leukemias, whereas genes involved in transport/metabolism were only upregulated in the CD34‐negative leukemias (Table S3).

Using the differentially expressed genes as seed proteins in a PPI network analysis revealed 10 networks significantly altered in CD34‐positive vs CD34‐negative leukemias (Table S4 and Figs S6–S15). Subjecting the proteins involved in these networks to overrepresentation analysis (GO, biological process) revealed key biological processes to include cell migration and adhesion, cellular response to stress, regulation of apoptosis, cell cycle, and IL7‐ and TGF‐beta signaling (Table S4). In particular, cell migration/adhesion and regulation of apoptosis were represented with high significance in several networks (Fig. 3C shows the representation of cell adhesion and migration in Network 5). To determine the overall direction of these processes, we looked at the full set of examined genes with a log2FC above 0.1 and categorized the genes within each overrepresented GO biological process as upregulated in CD34 positive or CD34 negative (Fig. 3D). The CD34‐positive leukemias showed upregulation of genes involved in cytokine signaling, positive regulation of cell migration and cell adhesion, TGF‐beta signaling, axonal guidance, and negative regulation of apoptosis. Contrarily, the CD34‐negative leukemias had upregulation of genes involved in cell cycle, cytoskeletal organization, positive regulation of apoptosis, protein transport, and cellular response to stress. Of note, these results were confirmed by GSEA, showing enrichment in CD34‐negative leukemias of, for example, several cell cycle‐related pathways, while CD34‐positive leukemias had enrichment of both integrin‐ and nonintegrin‐mediated cell adhesion (Fig. S16), positive regulation of cell adhesion, leukocyte migration, and negative regulation of apoptosis (Fig. S5B–D).

### 3.5 Ph‐like gene expression is identified in one‐third of CD34‐positive patients with relapse

The PPI network analysis (Network 9; Fig. S14) showed overrepresentation of genes involved in JAK‐STAT signaling, known to be constitutively activated in Ph‐like ALL [42]. Therefore, we investigated if the different outcomes of CD34‐positive and CD34‐negative patients within the B‐other subgroup could be driven by Ph‐like cases, as these are reported to be CD34‐positive and have a poor prognosis [7, 43]. Unlike most other BCP‐ALL subtypes, Ph‐like ALL is defined by the gene expression profile and represents a more genetically heterogeneous disease [42]. Out of 160 patients with available gene expression data, we were able to identify 10 patients (6.3%) with a Ph‐like gene expression profile. These patients were all CD34‐positive (PN, 2/10 with bimodal expression, full LAIP in Table S2). Of the 10 Ph‐like patients, only three had EOI MRD > 10^−3^, but five experienced relapse, comprising 36% of the CD34‐positive patients with relapse and available gene expression data. Of these, four had available flow cytometry data at time of relapse, two of whom had developed a CD34PB immunophenotype.

## 4 Discussion

In this study, we demonstrate for the first time a clear inverse association between CD34 surface expression and therapy response in Ph‐neg BCP‐ALL, independently of known risk factors.

The LAIP has been investigated in relation to prognosis in a variety of studies [6, 10, 44, 45, 46] but frequently with discrepant findings, as seen for CD20 [9, 11, 12, 47]. Discrepant results could be due to differences in FCM sensitivity, cytogenetic composition of the cohorts, and varying therapy regimes. Likewise, for CD34, one early study of mixed ALL, including 18 Ph+ BCP, 33 Ph− BCP, and 24 T‐ALL patients, found an overall association between CD34 expression and EOI MRD, but also found a higher prevalence of CD34 positivity in the Ph+ group [48]. Two other early studies suggested an association between CD34‐positive BCP‐ALL and a favorable outcome [49, 50] but were limited by reduced sensitivity due to few FCM‐acquired cells and/or hemodilution [49], as well as lack of cytogenetic analysis for rearrangements involving *KMT2A* [49, 50]. *KMT2A*‐r BCP‐ALL, more common in infants, has a very poor prognosis and often displays a CD10negCD20neg LAIP with around 50% CD34‐negative cases [51, 52]. Thus, the poor prognosis of *KMT2A*‐r could mask an inverse association of CD34 with therapy response in non‐*KMT2A*‐r patients. Our cohort only included four cases of *KMT2A*‐r ALL with equally high MRD levels in CD34 positive and CD34 negative, and our findings thus identify non‐*KMT2A*‐r CD34‐negative/predominantly negative cases as a subgroup with a very good response to induction therapy.

*IKZF1* alterations are seen in 70% of Ph‐like BCP‐ALL and loss of *IKZF1* function in combination with activated tyrosine kinase signaling, increased IL7R/CRLF2 signaling, and/or JAK/STAT signaling is known to associate with a poor outcome [53]. Interestingly, CD34 has been suggested a direct regulatory target of Ikaros [54] with WT Ikaros inducing downmodulation of surface CD34 in Ph+ BCP‐ALL [54]. In line with these prior findings, our PPI network analysis showed overrepresentation of genes involved in JAK‐STAT signaling in CD34‐positive leukemias, and further investigation revealed a Ph‐like profile among one‐third of patients with a CD34‐positive LAIP and relapse. It is not known whether the CD34 expression is an epiphenomenon associated with *IKZF1* alterations or plays an active role in the development of the comparatively more aggressive Ph‐like leukemias. *IKZF1* alterations have been shown to contribute to a stem‐like, glucocorticoid‐resistant, and adhesive phenotype with increased expression of, that is, integrin alpha 5 and L‐selectin in Ph+ BCP‐ALL [55], which is in line with our findings of upregulation of cell adhesion‐related genes in CD34‐positive leukemias and poor outcome of the CD34‐positive, Ph‐like patients. Also in line with our data is a study of 191 high‐risk BCP‐ALL cases, of which 56 harbored *IKZF1* alterations [56], showing high CD34 gene expression to associate with detectable MRD levels [54], while Cas9/CRISPR‐mediated depletion of CD34 resulted in reduced growth in liquid culture of *IKZF1*‐mutated human BCP‐ALL cells [54]. These previous works and our present study suggest a role for CD34 in the downstream effects of *IKZF1* alterations, but functional studies are needed to elucidate the actual role of CD34.

Our finding of an association between a CD34‐positive CD38 dim‐positive LAIP and poor therapy response raises the question whether this LAIP represents a less differentiated, stem‐like, and therapy‐resistant leukemic phenotype, as seen for CD34‐positive CD38‐negative leukemic cells in AML [57]. In this study, we were unable to find a tight relationship between CD34‐positive ALL and normal human HSCs. Several potential reasons exist why this may not be the case. Firstly, a consistent HSC profile has proven challenging to define [58], and secondly, activated rather than quiescent HSCs would be expected to be more similar to cancer stem cells. Finally, major differences might exist between AML and ALL in terms of what constitutes stem‐like, leukemia‐initiating cells. In ALL, leukemia‐initiating capacity has been shown for various immunophenotypes [59, 60] and therapy resistance and quiescence are thought to be reversible traits in ALL cell subsets, induced/maintained by the bone marrow microenvironment [61, 62], rather than inherent traits in dedicated leukemic stem cells *per se* [60, 61]. Despite not finding an overall difference in HSC‐associated genes between CD34‐positive and CD34‐negative samples, we did find enrichment in CD34‐positive leukemias of HSC‐associated genes in a rank‐based GSEA as well as upregulation of key individual genes, previously described to be associated with HSC in several studies, such as the gene encoding the growth guidance receptor ROBO4, which is expressed on HSC as well as AML LSC [63], and plays a role in healthy HSC trafficking [64, 65, 66, 67]. Also, the integrin alpha 6 gene (*ITGA6*, encoding CD49f), shown in several studies to be upregulated in HSC [68, 69, 70], playing an important role in HSC homing to the BM niche [71], and associating with poor therapy response in ALL [20], was among the top 5 upregulated genes in the CD34‐positive leukemias. Apart from a role in stem cell biology, CD34 is thought to play a dual role in adhesion/migration, where it on the one hand prevents homotypic cell‐cell adhesions, but on the other hand leads to basolateral membrane polarization of adhesion molecules such as integrins, increasing cellular adhesion to extracellular matrix. Further, CD34 enhances HSPC adhesion to endothelial cells of the BM sinusoidal vessels. In leukemia, a CD38‐negative CD34‐positive phenotype has been associated with increased adherence, dormancy, stem‐like features, and therapy resistance [25, 26, 27]. In our work, the most significant networks associated CD34 positive with migration, adhesion, and survival, thereby corroborating these earlier findings made in AML or based on ALL cell lines. Like CD34‐positive leukemia‐initiating AML cells [63] we find *ROBO*4 significantly overexpressed in CD34‐positive ALL samples, but we also find other axonal growth guidance genes upregulated, such as *EFNB1* and *EPHA7*. This is a particularly intriguing finding given the known roles of these genes in migration, adhesion, survival, and stemness [72, 73], yet functional studies are warranted to elucidate the exact roles of these genes in CD34‐positive ALL.

## 5 Conclusions

In conclusion, the present work shows that immunophenotype predicts therapy response in BCP‐ALL, independently of known risk factors. A CD34‐negative LAIP was associated with a good induction therapy response, although larger studies are needed to establish its direct significance for relapse risk and thus its potential as a risk stratification parameter. In contrast, high CD34 expression was associated with poor therapy response and with an abundance of genes involved in cell migration and adhesion, axonal guidance, TGF‐beta signaling, and negative regulation of apoptosis as well as decreased expression of cell cycle genes. In this study, we used a PPI network approach, allowing for a purely data‐driven, unbiased identification of key processes and networks separating leukemic subgroups, which could then subsequently be superimposed with annotations of biological processes and individual gene expression. It is, however, important to acknowledge that factors such as post‐transcriptional modifications and cellular/tissue localization complicate the direct extrapolation from predicted to *in vivo* interactions, and so functional studies are needed to confirm these predicted associations. In addition, gene expression profiles could be influenced by CG, as suggested for the Ph‐like subgroup. Finally, whether the identified key cellular processes vary among cell subsets within individual leukemias is yet to be determined, and further studies, such as deep phenotyping at single‐cell level of the leukemia at diagnosis and MRD timepoints, could provide valuable insights into the relationship between leukemic heterogeneity and therapy resistance.

## Conflict of interest

The authors declare no conflict of interest.

## Author contributions

HVM and SM conceptualized the study. HVM, NFØ, and GWJ performed the flow cytometric analysis, and GWJ provided the control bone marrow samples. SM and HVM reviewed flow files for scoring of marker expression. KS, BKA, SR, and PSW included the patients and provided clinical data. HOM performed PCR‐MRD analyses. Statistical analyses were performed by SM in collaboration with SR. LRO and MD analyzed the gene expression data, while RW performed the PPI network analysis. SM, CC, HVM, and RW performed the interpretation of the gene expression data and the PPI networks. TF and HL performed the identification of Ph‐like cases. The manuscript was written by SM with contributions from CC and HVM. All authors read and approved the final manuscript.

## Peer Review

The peer review history for this article is available at https://publons.com/publon/10.1002/1878‐0261.13207.

## Supporting information


**Fig. S1.** Gating strategy for nonmalignant B‐lymphopoiesis in the bone marrow.
**Fig. S2.** Immunophenotypic heterogeneity in BCP‐ALL.
**Fig. S3.** Association between gene expression and protein expression for immunophenotypic markers in BCP‐ALL.
**Fig. S4.** Principal component analysis of CD34‐positive and CD34‐negative cases.
**Fig. S5.** Gene set enrichment analysis of HSC signature genes and GO biological processes.
**Figs S6–S15.** Significantly altered PPI networks between CD34‐positive and CD34‐negative leukemias.
**Fig. S16.** Top ten enriched pathways in CD34‐negative and CD34‐positive leukemias.
**Table S1.** Reference intervals for immunophenotypic markers.
**Table S2A.** Immunophenotype by cytogenetic subgroup in BCP‐ALL.
**Table S2B.** CD34 expression by cytogenetic subgroup in BCP‐ALL patients with and without gene expression profiling data.
**Table S3.** Top 50 differentially expressed genes among CD34‐positive and CD34‐negative leukemias.
**Table S4.** Significant PPI networks between CD34‐positive and CD34‐negative leukemias.
**Data S1.** List of supplementary references.

## Data Availability

Research data are not shared due to ethical restrictions.
