# Supplementary material for: High CD34 surface expression in BCP‐ALL predicts poor induction therapy response and is associated with altered expression of genes related to cell migration and adhesion
Source: Mol Oncol. 2022 Apr 7;16(10):2015–30. doi: 10.1002/1878-0261.13207 (PMC9120905; doi:10.1002/1878-0261.13207)
Supplement: Supplementary file 1 — Fig. S1. Gating strategy for nonmalignant B‐lymphopoiesis in the bone marrow. Fig. S2. Immunophenotypic heterogeneity in BCP‐ALL. Fig. S3. Association between gene expression and protein expression for immunophenotypic markers in BCP‐ALL. Fig. S4. Principal component analysis of CD34‐positive and CD34‐negative cases. Fig. S5. Gene set enrichment analysis of HSC signature genes and GO biological processes. Figs S6–S15. Significantly altered PPI networks between CD34‐positive and CD34‐negative leukemias. Fig. S16. Top ten enriched pathways in CD34‐negative and CD34‐positive leukemias. Table S1. Reference intervals for immunophenotypic markers. Table S2A. Immunophenotype by cytogenetic subgroup in BCP‐ALL. Table S2B. CD34 expression by cytogenetic subgroup in BCP‐ALL patients with and without gene expression profiling data. Table S3. Top 50 differentially expressed genes among CD34‐positive and CD34‐negative leukemias. Table S4. Significant PPI networks between CD34‐positive and CD34‐negative leukemias. Data S1. List of supplementary references. [file MOL2-16-2015-s001.docx]

**Supplemental Material**

| CD marker | Neg | PD | PN | PB |
| --- | --- | --- | --- | --- |
| CD45 | isotype | - | isotype < and < upper limit preB II | > preB II |
| CD19 | T-cells | T-cells < and < preB I+II | preB I+II | > preB I+II |
| CD34 | preB II | - | preB I | > pre-B I |
| CD10 | T-cells | T-cells < and < preB II | pre-B I+II | > pre-B I |
| CD20 | T-cells | - | T-cells < and < mature B | > lower limit mature B |
| CD38 | Isotype | Isotype < and < preB I-III | preB I-III | > preB I-III (highest) |
| CD22 | T-cells | - | preB I+II (widest) | > preBII |
| nTdT | T-cells | - | preB I | > preB I |
| CyCD22 | T-cells | - | preB I+II | > preB I+II |
| CyCD79a | T-cells | T-cells < and < 10^3^ | 10^3^-10^4^ | >10^4^ |
| CD123 | T-cells | T-cells <and < 10^3^ | 10^3^-10^4^ | >10^4^ |
| CD13 | T-cells | T-cells < and < 10^3^ | 10^3^-10^4^ | >10^4^ |
| CD66c | T-cells | T-cells < and < 10^3^ | 10^3^-10^4^ | >10^4^ |
| CD133 | T-cells | T-cells < and < 10^3^ | 10^3^-10^4^ | >10^4^ |
| CD33 | T-cells | T-cells < and < 10^3^ | 10^3^-10^4^ | >10^4^ |
| CD15 | T-cells | T-cells < and < 10^3^ | 10^3^-10^4^ | >10^4^ |

**Table S1** **Reference intervals for immunophenotypic markers**

Reference intervals for immunophenotypic characterization of BCP-ALL leukemic clones were based on surface marker expression levels on mature B-cells, B-cell precursors (stage I-III) and T-cells from unaffected bone marrow samples (Figure S1). For cross-lineage expressed markers, decades were used to define limits when appropriate. For markers, where the expression on normal B-cell precursors is dim (CD34, CD22, nTdT, cyCD22), PN was defined based on normal BCP and so no PD area was defined. Populations were furthermore defined as PD for any unimodally expressed marker, when the majority of cells were negative, but more than 20% of the population was brighter than the neg area. Neg: Negative, PD: Positive dim, PN: Positive normal, PB: Positive bright.

|  | **HeH** | **T(12;21)** | **T(1;19)** | **IAMP21** | **Dic(9;20)** | **KMT2A** | **Hypodiploid** | **Ph-like** | **B-other** | **Total** |
| --- | --- | --- | --- | --- | --- | --- | --- | --- | --- | --- |
| **CD34** | 2/2/41/2 (15) | 1/1/12/2 (19) | 5/0/0/0  (0) | 0/0/3/0  (1) [1] | 1/0/0/0 (1) | 2/0/0/0 (2) | 1/1/1/1  (2) | 0/0/8/0 (2) | 10/0/34/5 (22) | 22/4/99/10  (64) [1] |
| **CD38** | 0/32/28/0 (2) | 0/16/18/0 (1) | 0/1/4/0  (0) | 0/4/0/0  (1) | 0/2/0/0 (0) | 0/1/3/0 (0) | 0/2/4/0  (0) | 0/7/3/0 (0) | 0/19/46/1 (4) [1] | 0/82/108/1  (8) [1] |
| **CD10** | 0/2/14/45 (1) | 0/1/2/28 (4) | 0/0/4/0  (0) [1] | 0/0/0/4  (1) | 0/0/1/1 (0) | 3/0/1/0 (0) | 0/0/5/1  (0) | 0/0/1/8 (1) | 7/10/14/33 (7) | 10/13/42/120  (14) [1] |
| **CD20** | 20/20/15/3 (4) | 20/13/1/0 (1) | 4/0/0/0  (0) [1] | 1/2/1/1  (0) | 0/1/1/0 (0) | 4/0/0/0 (0) | 2/1/0/3  (0) | 1/4/5/0 (0) | 35/13/13/5 (5) | 87/54/36/12  (10) [1] |
| **CD19** | 0/0/61/0 (1) | 0/0/35/0 (0) | 0/0/5/0  (0) | 0/0/5/0  (0) | 0/0/2/0 (0) | 0/0/3/0 (1) | 0/0/6/0  (0) | 0/0/10/0 (0) | 0/0/66/0 (5) | 0/0/193/0  (7) |
| **CD45** | 17/25/15/0 (5) | 4/8/20/0 (1) [2] | 0/0/5/0  (0) | 0/0/5/0  (0) | 0/0/2/0  (0) | 0/0/4/0  (0) | 2/0/2/1  (1) | 0/0/8/0  (2) | 16/8/40/0 (7) | 39/41/101/1  (16) [2] |
| **CD22** | 0/1/58/1 (0) [2] | 0/2/32/1 (0) | 0/0/4/0 (0) [1] | 0/0/4/1  (0) | 0/0/2/0  (0) | 0/1/2/0  (0) [1] | 0/2/4/0  (0) | 0/0/8/2  (0) | 0/3/54/10 (4) | 0/9/168/15  (4) [4] |
| **nTdT** | 1/6/45/1  (8) [1] | 1/1/26/0  (7) | 0/0/4/0  (0) [1] | 0/0/5/0  (0) | 0/0/2/0  (0) | 0/2/2/0  (0) | 0/0/6/0  (0) | 0/0/8/0  (2) | 2/9/51/0  (7) [2] | 4/18/149/1  (24) [4] |
| **CyCD22** | 0/9/44/1  (4) [4] | 2/8/22/0  (1) [2] | 0/2/2/0  (0) [1] | 0/0/4/0  (1) | 0/1/1/0  (0) | 0/1/3/0  (0) | 0/1/5/0  (0) | 1/2/5/1 (1) | 2/19/40/3  (4) [3] | 5/43/126/5  (11) [10] |
| **CyCD79a** | 0/1/52/2  (3) [4] | 0/6/22/4  (1) [2] | 0/0/4/0  (0) [1] | 0/0/4/0  (1) | 0/0/2/0  (0) | 0/1/2/0  (1) | 0/0/5/1  (0) | 0/0/6/2 (2) | 1/3/49/9  (6) [3] | 1/11/146/18  (14) [10] |
| **CD133** | 9/36/14/0  (0) [3] | 26/8/1/0  (0) | 5/0/0/0  (0) | 0/3/2/0  (0) | 2/0/0/0  (0) | 2/0/2/0  (0) | 6/0/0/0  (0) | 4/4/2/0  (0) | 41/21/4/0  (3) [2] | 95/72/25/0  (3) [5] |
| **CD13** | 34/22/1/0  (0) [5] | 8/10/15/0  (0) [2] | 2/3/0/0  (0) | 3/2/0/0  (0) | 2/0/0/0  (0) | 3/0/0/0  (0) [1] | 3/3/0/0  (0) | 5/4/1/0 (0) | 39/23/4/0  (2) [3] | 99/67/21/0  (2) [11] |
| **CD123** | 0/0/43/16  (1) [2] | 1/20/14/0  (0) | 2/3/0/0  (0) | 0/3/2/0  (0) | 0/2/0/0  (0) | 0/1/2/0  (1) | 2/1/2/1  (0) | 0/6/4/0 (0) | 5/32/29/3  (2) | 10/68/96/20  (4) [2] |
| **CD66c** | 2/20/23/9  (7) [1] | 29/5/1/0  (0) | 4/0/0/0  (0) [1] | 0/2/1/2  (0) | 0/0/1/0  (1) | 4/0/0/0  (0) | 1/4/0/0  (1) | 1/6/2/1 (0) | 41/13/10/3  (3) [1] | 82/50/38/15  (12) [3] |
| **CD33** | 56/2/0/0  (0) [4] | 15/16/2/0  (0) [2] | 5/0/0/0  (0) | 3/2/0/0  (0) | 1/1/0/0  (0) | 3/0/0/0  (0) [1] | 6/0/0/0  (0) | 9/1/0/0 (0) | 45/21/3/0  (0) [2] | 143/43/5/0  (0) [9] |
| **CD15** | 52/6/0/0  (0) [4] | 32/1/0/0  (1) [1] | 4/1/0/0  (0) | 5/0/0/0  (0) | 1/1/0/0  (0) | 1/1/0/0  (1) [1] | 6/0/0/0  (0) | 10/0/0/0 (0) | 57/9/1/0  (0) [4] | 168/19/1/0  (2) [10] |

**Table S2A** **Immunophenotype by cytogenetic subgroup in BCP-ALL**

Patient counts are given for unimodally negative/PD/PN/PB followed by the number of patients with bimodal expression in parenthesis and missing data, if any, in brackets. Two patients with iAMP21 and two patients with high hyperdiploidy were classified as Ph-like by their gene expression profile and were thus counted in the Ph-like group. N(HeH)=62, n(t(12;21))=35, n(t(1;19)=5, n(iAMP21)=5, n(dic(9;20))=2, n(KMT2A)=4, n(hypodiploid)=6, n(Ph-like)=10, n(B-other)=71. PD: Positive dim, PN: Positive normal, PB: Positive bright.

|  | **HeH** | **T(12;21)** | **T(1;19)** | **IAMP21** | **Dic(9;20)** | **KMT2A** | **Hypodiploid** | **Ph-like** | **B-other** | **Total** |
| --- | --- | --- | --- | --- | --- | --- | --- | --- | --- | --- |
| **GEP** | 49 | 27 | 3 | 4 | 2 | 4 | 4 | 10 | 57 | 160 |
| **GEP by CD34** | 1/2/34/1 (11) | 1/1/9/1 (15) | 3/0/0/0 (0) | 0/0/3/0  (1) | 1/0/0/0  (1) | 2/0/0/0  (2) | 0/1/1/0  (2) | 0/0/8/0  (2) | 10/0/26/4  (17) | 18/4/81/6  (51) |
| **No GEP** | 13 | 8 | 2 | 1 | 0 | 0 | 2 | 0 | 14 | 40 |
| **No GEP by CD34** | 1/0/7/1  (4) | 0/0/3/1  (4) | 2/0/0/0  (0) | 0/0/0/0  (0) [1] | 0/0/0/0  (0) | 0/0/0/0  (0) | 1/0/0/1  (0) | 0/0/0/0  (0) | 0/0/8/1  (5) | 4/0/18/4  (13) [1] |

**Table S2B** **CD34 expression by cytogenetic subgroup in BCP-ALL patients with and without gene expression profiling data**

Patient counts are given for unimodally negative/PD/PN/PB followed by the number of patients with bimodal expression in parenthesis and missing data, if any, in brackets. Two patients with iAMP21 and two patients with high hyperdiploidy were classified as Ph-like by their gene expression profile and were thus counted in the Ph-like group. N(HeH)=62, n(t(12;21))=35, n(t(1;19)=5, n(iAMP21)=5, n(dic(9;20))=2, n(KMT2A)=4, n(hypodiploid)=6, n(Ph-like)=10, n(B-other)=71. GEP: Gene expression profiling, HeH: High hyperdiploidy, PD: Positive dim, PN: Positive normal, PB: Positive bright.

| **Upregu-lation** | **Uniprot ID** | **Ensemble ID** | **Log2FC** | **Q value** | **GO proces** | **Category** | **Associated with stemness** |
| --- | --- | --- | --- | --- | --- | --- | --- |
| CD34pos | S10AG_HUMAN | ENSG00000188643 | -3.52 | 4.66E-07 | GO:0051592 | signaling | [1] |
| CD34pos | TSN7_HUMAN | ENSG00000156298 | -2.26 | 9.84E-04 | GO:0016032 | immunological function | [2] |
| CD34pos | ENTK_HUMAN | ENSG00000154646 | -2.23 | 1.47E-02 | GO: 0006508 | proteolysis | No |
| CD34pos | DAPK1_HUMAN | ENSG00000196730 | -1.92 | 1.58E-05 | GO:0006915 | apoptosis/survival | [3] |
| CD34pos | ITA6_HUMAN | ENSG00000091409 | -1.82 | 3.60E-04 | GO:0007155 | cell adhesion/migration/ cytoskeletal dynamics | [4] |
| CD34pos | CD109_HUMAN | ENSG00000156535 | -1.79 | 1.43E-06 | GO:0010466 | proteolysis | [5] |
| CD34pos | IF44L_HUMAN | ENSG00000137959 | -1.77 | 2.82E-02 | GO:0006955 | immunological function | [6] |
| CD34pos | CYTL1_HUMAN | ENSG00000170891 | -1.69 | 3.60E-04 | GO:007165 | signaling | [7] |
| CD34pos | 3BP5_HUMAN | ENSG00000131370 | -1.69 | 1.15E-03 | GO:007165 | signaling | No |
| CD34pos | CCND2_HUMAN | ENSG00000118971 | -1.64 | 9.00E-06 | GO:0051726 | proliferation | [8] |
| CD34pos | MRC1_HUMAN | ENSG00000260314 | -1.59 | 1.95E-03 | GO:0016032 | immunological function | [9] |
| CD34pos | I13R1_HUMAN | ENSG00000131724 | -1.51 | 2.81E-03 | GO:0019221 | signaling | [10] |
| CD34pos | HBEGF_HUMAN | ENSG00000113070 | -1.49 | 2.81E-03 | GO:007165 | signaling | [11] |
| CD34pos | SI1L2_HUMAN | ENSG00000116991 | -1.48 | 6.59E-04 | GO:0051056 | signaling | No |
| CD34pos | EPHA7_HUMAN | ENSG00000135333 | -1.47 | 1.22E-02 | GO:0022407 | cell adhesion/migration/ cytoskeletal dynamics | [12] |
| CD34pos | PO4F1_HUMAN | ENSG00000152192 | -1.46 | 2.17E-02 | GO:0010628 | transcription | [13] |
| CD34pos | LGMN_HUMAN | ENSG00000100600 | -1.45 | 1.40E-03 | GO: 0006508 | proteolysis | [14] |
| CD34pos | EFNB1_HUMAN | ENSG00000090776 | -1.44 | 9.44E-05 | GO:0007155 | cell adhesion/migration/ cytoskeletal dynamics | [15] |
| CD34pos | SNAI1_HUMAN | ENSG00000124216 | -1.41 | 3.07E-03 | GO:0006357 | transcription | [16] |
| CD34pos | RFLB_HUMAN | ENSG00000183688 | -1.38 | 1.99E-04 | GO:0030036 | cell adhesion/migration/ cytoskeletal dynamics | No |
| CD34pos | FA49A_HUMAN | ENSG00000197872 | -1.36 | 6.08E-06 | GO:0030833 | cell adhesion/migration/  cytoskeletal dynamics | No |
| CD34pos | EMP1_HUMAN | ENSG00000134531 | -1.34 | 3.02E-02 | GO:0008219 | apoptosis/survival | [17] |
| CD34pos | TLR6_HUMAN | ENSG00000174130 | -1.34 | 3.09E-03 | GO:0006955 | immunological function | [18] |
| CD34pos | PLCH1_HUMAN | ENSG00000114805 | -1.31 | 1.59E-02 | GO:0007165 | signaling | No |
| CD34pos | IL3RA_HUMAN | ENSG00000185291 | -1.30 | 6.85E-03 | GO:0019221 | signaling | No |
| CD34pos | TM236_HUMAN | ENSG00000148483 | -1.29 | 3.20E-02 | N.A. | not annotated | No |
| CD34neg | BEST3_HUMAN | ENSG00000127325 | 2.37 | 3.60E-04 | GO:0006811 | transport and metabolism | No |
| CD34neg | LAMP5_HUMAN | ENSG00000125869 | 2.13 | 1.39E-06 | GO:0072594 | miscellaneous | [19] |
| CD34neg | ELOV2_HUMAN | ENSG00000197977 | 2.08 | 1.08E-05 | GO:0000038 | transport and metabolism | No |
| CD34neg | NRG3_HUMAN | ENSG00000185737 | 2.05 | 2.44E-07 | GO:0035556 | signaling | [20] |
| CD34neg | VAT1L_HUMAN | ENSG00000171724 | 1.90 | 1.59E-06 | N.A. | not annotated | No |
| CD34neg | NID2_HUMAN | ENSG00000087303 | 1.89 | 2.54E-06 | GO:0007155 | cell adhesion/migration/ cytoskeletal dynamics | [21] |
| CD34neg | RAG2_HUMAN | ENSG00000175097 | 1.84 | 3.89E-02 | GO:0033151 | immunological function | No |
| CD34neg | S27A2_HUMAN | ENSG00000140284 | 1.73 | 1.24E-06 | N.A. | not annotated | No |
| CD34neg | AT1A3_HUMAN | ENSG00000105409 | 1.69 | 8.29E-06 | GO:0006811 | transport and metabolism | No |
| CD34neg | KCNQ5_HUMAN | ENSG00000185760 | 1.68 | 5.96E-06 | GO:0006811 | transport and metabolism | No |
| CD34neg | NCKP5_HUMAN | ENSG00000176771 | 1.67 | 7.40E-06 | GO:0007019 | miscellaneous | No |
| CD34neg | FAT1_HUMAN | ENSG00000083857 | 1.61 | 5.38E-03 | GO:0007155 | cell adhesion/migration/ cytoskeletal dynamics | [22] |
| CD34neg | PAWR_HUMAN | ENSG00000177425 | 1.54 | 2.30E-05 | GO:0006915 | apoptosis/survival | [23] |
| CD34neg | SPAG6_HUMAN | ENSG00000077327 | 1.53 | 4.68E-05 | GO:0030030 | cell adhesion/migration/ cytoskeletal dynamics | No |
| CD34neg | NETO2_HUMAN | ENSG00000171208 | 1.51 | 5.47E-04 | GO:2000312 | not annotated | [24] |
| CD34neg | I12R2_HUMAN | ENSG00000081985 | 1.51 | 4.66E-07 | GO:0019221 | signaling | No |
| CD34neg | TCL6 | ENSG00000187621 | 1.46 | 4.69E-03 | N.A. | not annotated | No |
| CD34neg | RED1_HUMAN | ENSG00000197381 | 1.44 | 4.66E-07 | GO:0006396 | miscellaneous | No |
| CD34neg | IKZF2_HUMAN | ENSG00000030419 | 1.42 | 2.31E-04 | GO:0006357 | transcription | [25] |
| CD34neg | MYEF2_HUMAN | ENSG00000104177 | 1.35 | 4.04E-03 | GO:0006357 | transcription | No |
| CD34neg | FCRLA_HUMAN | ENSG00000132185 | 1.35 | 1.05E-06 | GO:0007166 | signaling | No |
| CD34neg | TCL1B_HUMAN | ENSG00000213231 | 1.30 | 8.63E-04 | GO:0071902 | signaling | No |
| CD34neg | MCTP2_HUMAN | ENSG00000140563 | 1.30 | 6.89E-03 | GO:0019722 | signaling | No |
| CD34neg | ROR1_HUMAN | ENSG00000185483 | 1.28 | 9.99E-06 | GO:0033674 | signaling | [26] |

**Table S3** **Top 50 differentially expressed genes among CD34 positive and CD34 negative leukemias**

Top 50 differentially expressed genes as measured by log2 fold change among genes significant after FDR correction (q value <0.05). The CD34 gene itself was the most differentially expressed but was omitted from this analysis. Genes were characterized using the gene ontology biological process annotation according to the Uniprot database (www.uniprot.org) and compiled into categories where possible. Further, a thorough literature search was performed to identify associations with stemness (including hematopoietic stem cells, other types of stem cells or cancer-initiating stem cells). Numbers in brackets are supplementary references.

| Network | Iterations | Effective size | NW integrated p-value | NW central gene | GO term | GO ID | Genes in network | Enrich-ment | FDR adjusted p-value |
| --- | --- | --- | --- | --- | --- | --- | --- | --- | --- |
| 1 | 155/10e7 | 47 | 4.3e-13 | RUNX3 | Regulation of transcription by RNA polymerase II | GO:0006357 | 40 | 12.51 | 2.00E-38 |
|  |  |  |  |  | Positive regulation of Notch signaling pathway | GO:0045747 | 11 | 99.99 | 6.20E-22 |
|  |  |  |  |  | Response to hypoxia | GO:0001666 | 14 | 30.42 | 1.40E-17 |
|  |  |  |  |  | Beta-catenin-TCF complex assembly | GO:1904837 | 9 | 99.99 | 2.50E-17 |
|  |  |  |  |  | Regulation of cell differentiation | GO:0045595 | 19 | 10.73 | 2.70E-15 |
|  |  |  |  |  | Regulation of transforming growth factor beta receptor signaling pathway | GO:0017015 | 9 | 46.78 | 2.50E-13 |
| 2 | 323/10e7 | 24 | 2.5e-10 | CEP170 | Microtubule cytoskeleton organization | GO:0000226 | 10 | 27.64 | 1.10E-12 |
|  |  |  |  |  | Cell cycle | GO:0007049 | 12 | 8.07 | 5.90E-09 |
|  |  |  |  |  | G2/M transition of mitotic cell cycle | GO:0000086 | 5 | 31.12 | 5.00E-07 |
| 3 | 497/10e7 | 42 | 1.1e-11 | BRK1 | Fc-gamma receptor signaling pathway | GO:0038094 | 22 | 78.18 | 1.10E-37 |
|  |  |  |  |  | Regulation of cytoskeleton organization | GO:0051493 | 20 | 30.04 | 2.10E-25 |
|  |  |  |  |  | Vascular endothelial growth factor receptor signaling pathway | GO:0048010 | 9 | 64.94 | 1.10E-14 |
|  |  |  |  |  | Vesicle-mediated transport | GO:0016192 | 22 | 7.07 | 1.40E-14 |
|  |  |  |  |  | Cell adhesion | GO:0007155 | 13 | 9.13 | 7.20E-10 |
|  |  |  |  |  | Cell motility | GO:0048870 | 10 | 8.53 | 1.70E-07 |
| 4 | 498/10e7 | 75 | 2.2e-14 | GDI2 | Guanyl-nucleotide exchange factor activity | GO:0005085 | 27 | 40.3 | 8.70E-37 |
|  |  |  |  |  | Vesicle-mediated transport | GO:0016192 | 41 | 7.19 | 2.30E-26 |
|  |  |  |  |  | Secretion | GO:0046903 | 16 | 5.21 | 5.00E-08 |
|  |  |  |  |  | Regulation of Rab protein signal transduction | GO:0032483 | 3 | 99.99 | 2.20E-07 |
| 5 | 745/10e7 | 63 | 4.9e-13 | CD44 | Locomotion | GO:0040011 | 35 | 12.91 | 1.50E-30 |
|  |  |  |  |  | Cell migration | GO:0016477 | 28 | 14.26 | 2.90E-25 |
|  |  |  |  |  | Regulation of programmed cell death | GO:0043067 | 27 | 7.79 | 1.50E-17 |
|  |  |  |  |  | Positive regulation of cell migration | GO:0030335 | 18 | 13.21 | 1.20E-15 |
|  |  |  |  |  | Regulation of cell adhesion | GO:0030155 | 16 | 9.97 | 4.10E-12 |
|  |  |  |  |  | Regulation of response to stress | GO:0080134 | 20 | 5.45 | 3.30E-10 |
|  |  |  |  |  | Cell differentiation | GO:0030154 | 25 | 3.93 | 1.20E-09 |
|  |  |  |  |  | Negative regulation of cell migration | GO:0030336 | 5 | 7.1 | 7.00E-04 |
| 6 | 812/10e7 | 24 | 1.2e-9 | PLXNA2 | Neuron development | GO:0048666 | 11 | 47.17 | 9.30E-17 |
|  |  |  |  |  | Regulation of cell adhesion | GO:0030155 | 12 | 26.16 | 3.10E-15 |
|  |  |  |  |  | Regulation of actin cytoskeleton organization | GO:0032956 | 7 | 30.89 | 1.70E-09 |
|  |  |  |  |  | Axon guidance | GO:0007411 | 5 | 47.35 | 5.70E-08 |
| 7 | 825/10e7 | 74 | 1.2e-13 | RAC2 | Regulation of small GTPase mediated signal transduction | GO:0051056 | 34 | 15.95 | 3.90E-31 |
|  |  |  |  |  | Protein transport | GO:0015031 | 62 | 5.61 | 5.60E-30 |
|  |  |  |  |  | Actin cytoskeleton organization | GO:0030036 | 25 | 12.13 | 4.60E-20 |
|  |  |  |  |  | Secretion | GO:0046903 | 41 | 5.04 | 6.70E-18 |
|  |  |  |  |  | Vascular endothelial growth factor receptor signaling pathway | GO:0048010 | 15 | 22.28 | 1.40E-16 |
|  |  |  |  |  | Regulation of cell adhesion | GO:0030155 | 20 | 4.7 | 1.10E-08 |
| 8 | 874/10e7 | 25 | 9.6e-10 | CYTH2 | Insulin receptor signaling pathway | GO:0008286 | 7 | 99.99 | 3.40E-13 |
|  |  |  |  |  | Vesicle-mediated transport | GO:0016192 | 14 | 7 | 1.10E-09 |
| 9 | 938/10e7 | 180 | 4.8e-20 | JAK2 | Cytokine-mediated signaling pathway | GO:0019221 | 110 | 18.58 | 9.90E-99 |
|  |  |  |  |  | Positive regulation of tyrosine phosphorylation of STAT protein | GO:0042531 | 24 | 53.6 | 6.70E-37 |
|  |  |  |  |  | MAPK cascade | GO:0000165 | 41 | 13.08 | 6.30E-34 |
|  |  |  |  |  | Regulation of cell adhesion | GO:0030155 | 42 | 10.12 | 4.00E-30 |
|  |  |  |  |  | Response to stress | GO:0006950 | 88 | 3.61 | 1.60E-29 |
|  |  |  |  |  | Positive regulation of T cell activation | GO:0050870 | 26 | 22.33 | 5.50E-28 |
|  |  |  |  |  | Interleukin-7-mediated signaling pathway | GO:0038111 | 13 | 68.76 | 1.60E-22 |
|  |  |  |  |  | Negative regulation of programmed cell death | GO:0043069 | 38 | 7.4 | 2.50E-22 |
|  |  |  |  |  | Positive regulation of programmed cell death | GO:0043068 | 20 | 5.6 | 5.60E-10 |
| 10 | 944/10e7 | 160 | 1.2e-18 | NFKB1 | Regulation of transcription, DNA-templated | GO:0006355 | 97 | 6.04 | 3.90E-55 |
|  |  |  |  |  | Cellular response to stress | GO:0033554 | 54 | 5.17 | 1.00E-24 |
|  |  |  |  |  | Regulation of programmed cell death | GO:0043067 | 44 | 5.85 | 4.70E-22 |
|  |  |  |  |  | Regulation of cell differentiation | GO:0045595 | 39 | 6.86 | 6.20E-22 |
|  |  |  |  |  | Response to interleukin-1 | GO:0070555 | 21 | 18.49 | 6.70E-21 |
|  |  |  |  |  | Response to tumor necrosis factor | GO:0034612 | 17 | 11.44 | 1.50E-13 |

**Table S4** **Significant PPI networks between CD34 positive and CD34 negative leukemias.**

For each network, two types of information is provided: 1) Output from the statistical assessment of the network based on the gene expression data (column 1-5) and 2) An annotation of the biological function of the network based on Gene Ontology overrepresentation analysis of the genes/predicted proteins present in the network (column 6-10).
Key columns in section 1): Network (column 1): Name/rank of the network. Iterations (column 2): number of iterations out of 10^7^ where the permutated data had an equal or more extreme integrated p-value. Effective size (column 3): Number of nodes in the network that has a p-value in the differential expression data. NW integrated p-value (column 4): Edgington integration of these p-values. NW central gene (column 5): Network central gene/protein (notice, that all genes/proteins in the network contributed to the statistical assessment).
Key columns in section 2): Gene Ontology (GO) biological processes overrepresented in the given network are listed (columns 6+7). The number of genes annotated to the given GO biological process present in the corresponding network (column 8) as well as the enrichment score (column 9) and q-value for the process are given (column 10).

**
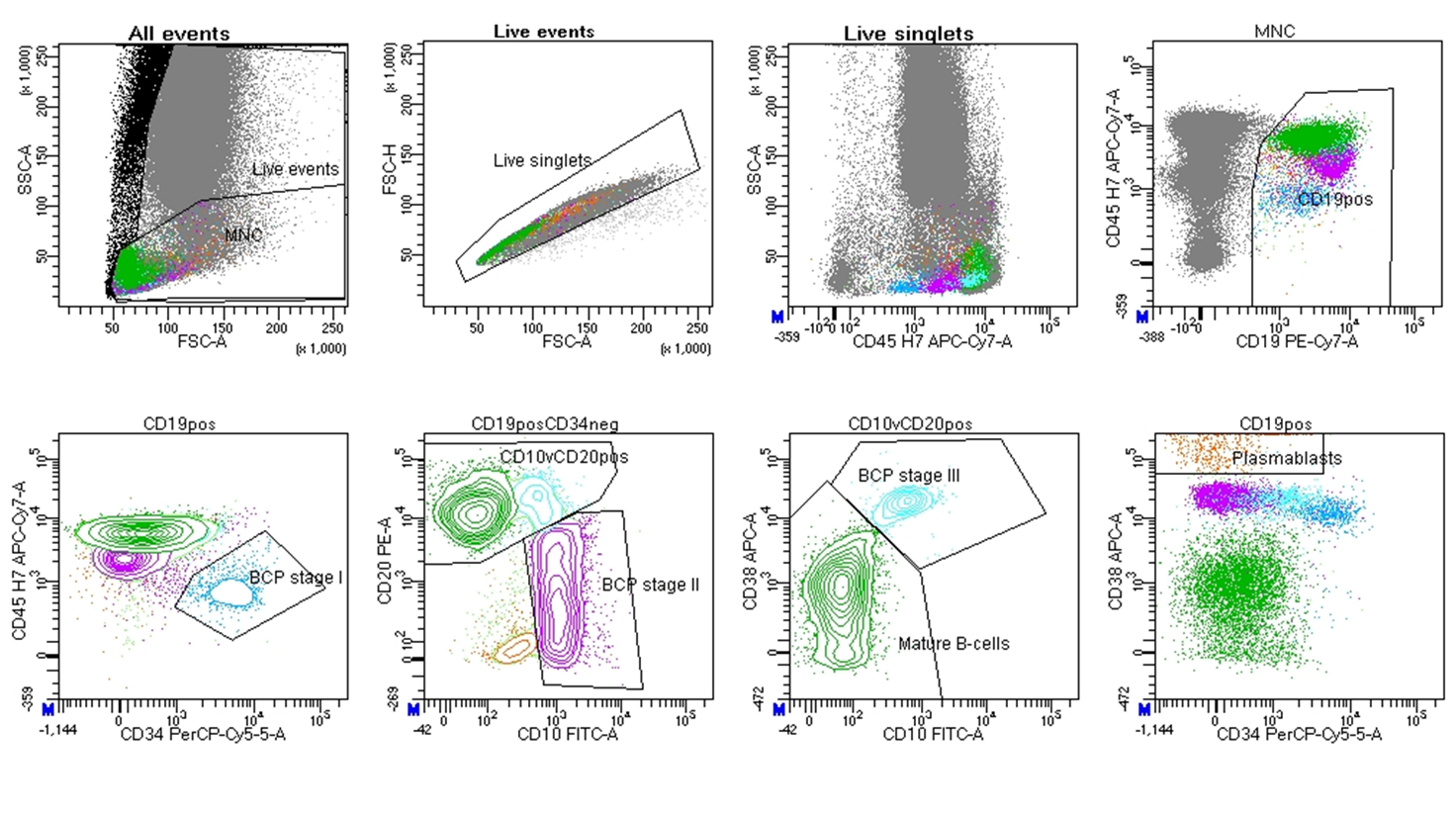
**

**Figure S1** **Gating strategy for non-malignant B-lymphopoiesis in the bone marrow**

Top row: Dead cells/debris and doublets are removed using the dot plots FSC/SSC and FSC Area/FSC Height. The CD45/SSC plot provides a useful overview of the gated populations and can be used as a visual check of the gated cells. The lineage marker CD19 is used as primary gating marker and selected on the CD45/ CD19 dot plot.

Bottom row: The CD34pos (stage I) B-cell precursors are gated in a CD45/CD34 contour plot below the CD19 gate in the hierarchy. Next, this gated is inverted (called CD19posCD34neg in this analysis), and the inverted gate is illustrated in a CD20/CD10 contour plot. The CD10posCD34neg (stage II) B-cell precursors are then gated, as well as the CD10vCD20posCD19posCD34neg cells, comprising transitional (stage III) and mature B-cells. These two populations are then separately gated in a CD38/CD10 contour plot. The plasmablasts are identified within the CD19pos cells as CD38high in a CD38/CD34 dot plot and should be verified as CD10negCD20neg in the CD20/CD10 contour plot.

For the definition of normal reference intervals, the upper and lower 10% contour limit fluorescence intensity levels of the identified populations (pre-B I,II,III, mature B, as well as CD3pos T-cells (not illustrated in the figure)) were identified in plots containing only the population of interest, in five bone marrow samples with unaffected B-lymphopoiesis, evenly distributed over the inclusion period. The median value of the five samples was used for defining the reference intervals (Table S1).

**
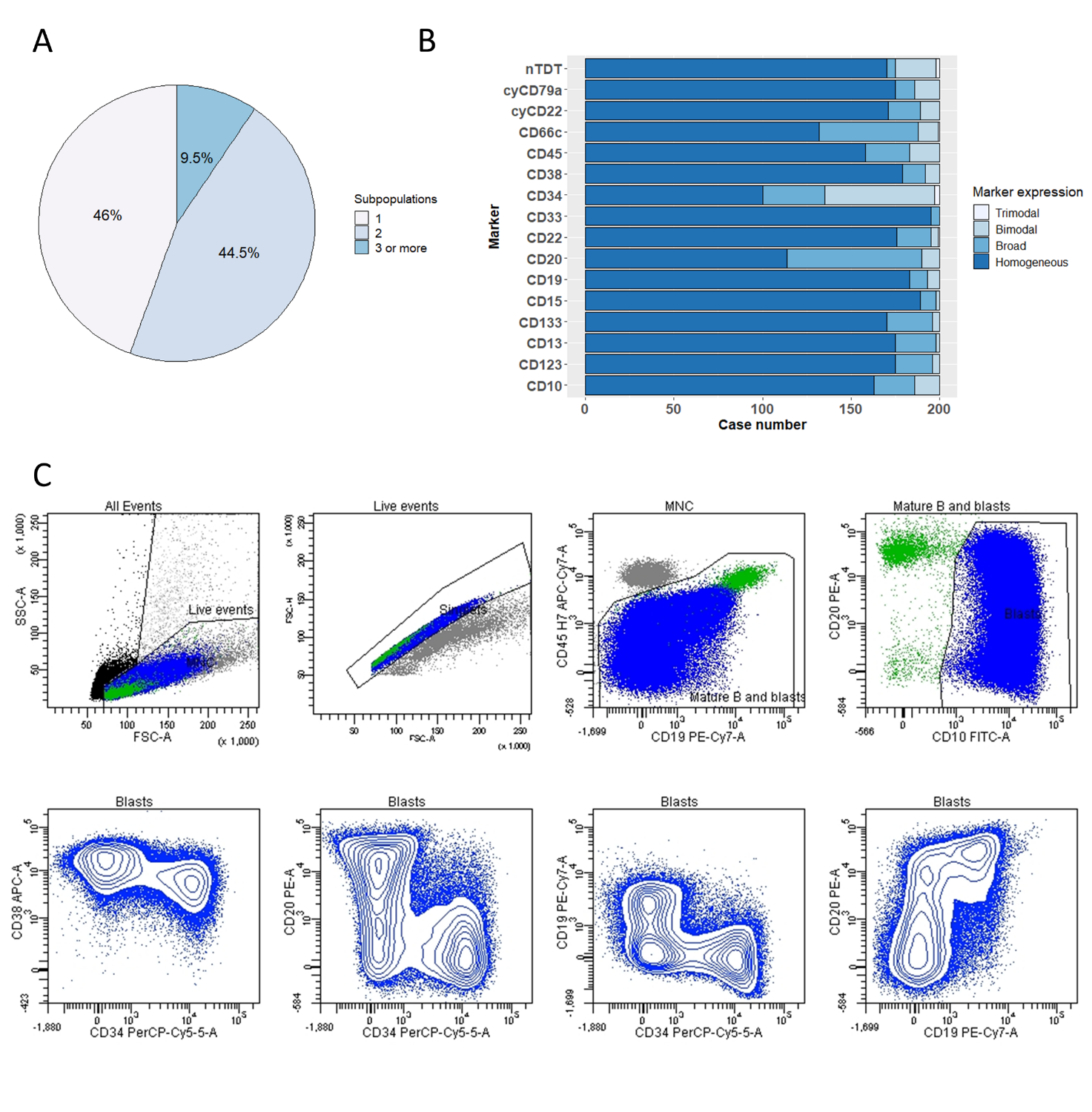
**

**Figure S2** **Immunophenotypic heterogeneity in BCP-ALL**

A: Distribution of immunophenotypic subpopulations at time of diagnosis among the 200 BCP-ALL cases. B: Distribution of homogeneous, heterogeneous broad, heterogeneous bimodal, and heterogeneous trimodal expression for each immunophenotypic marker. C: A BCP-ALL case with three subpopulations, illustrated in the CD20/CD34 plot, the CD19-CD34, and the CD20/CD19 plot.


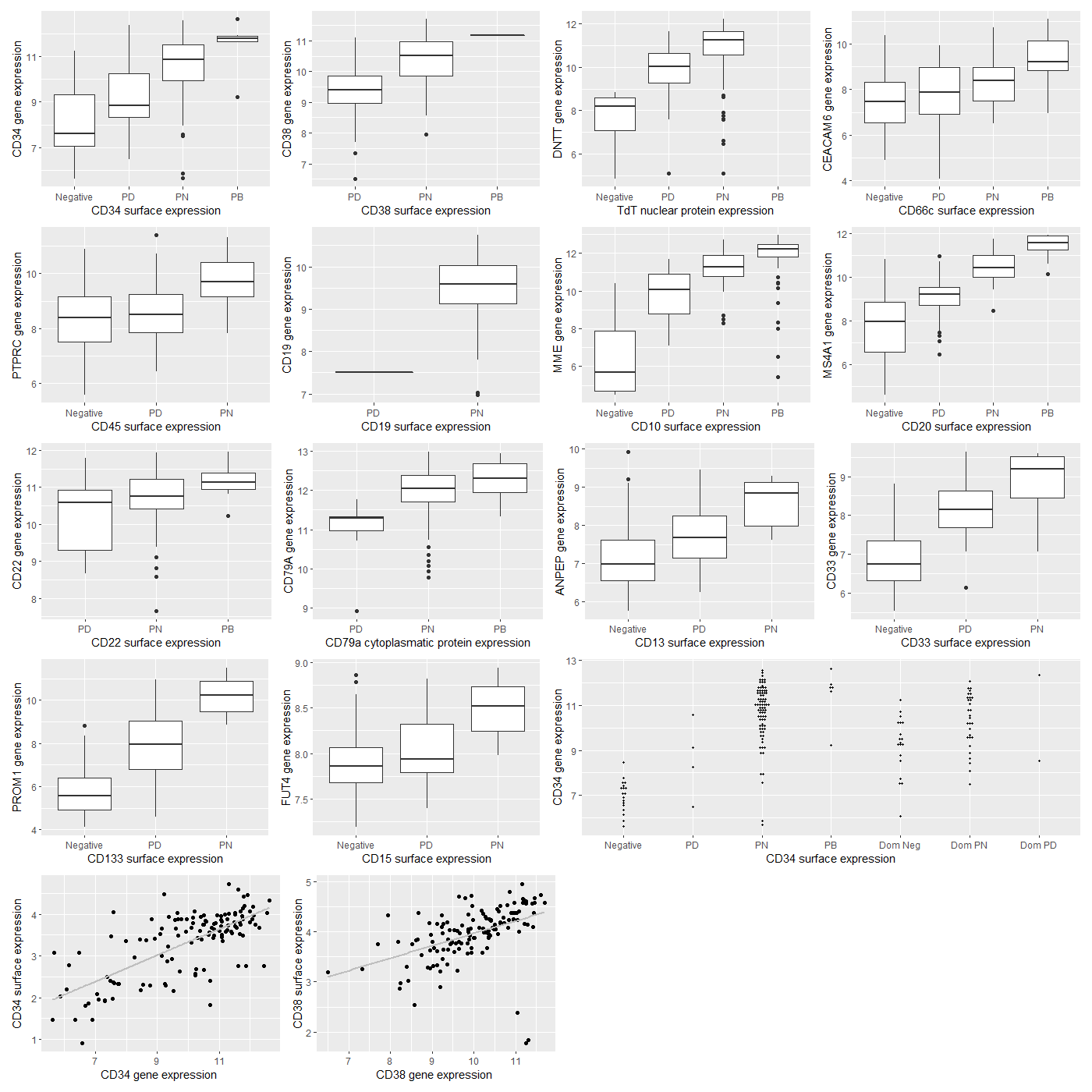


**Figure S3** **Association between gene expression and protein expression for immunophenotypic markers in BCP-ALL**

Normalized gene expression versus surface/cytoplasmatic (for CD79a)/nuclear (for nTdT) protein expression for the investigated immunophenotypic markers. Boxplots illustrate the combined score (Negative/PD/PN/PB), where cases with bimodal and unimodal expression have been pooled, according to the score of the predominant subpopulation in cases with bimodal expression and the score of the whole population in cases with unimodal expression. For CD34, a dot plot additionally illustrates separately cases with unimodal expression (Negative/PD/PN/PB) and bimodal expression (Dom Neg/Dom PN/Dom PD). Bottom row dot plots illustrate the association between surface expression (log10 of mean fluorescence intensity) and gene expression for CD34 and CD38 including a regression line. PD: Positive dim, PN: Positive normal, PB: Positive bright. Dom neg: Predominantly negative. Dom PD: Predominantly positive (dim). Dom PN: Predominantly positive (normal).


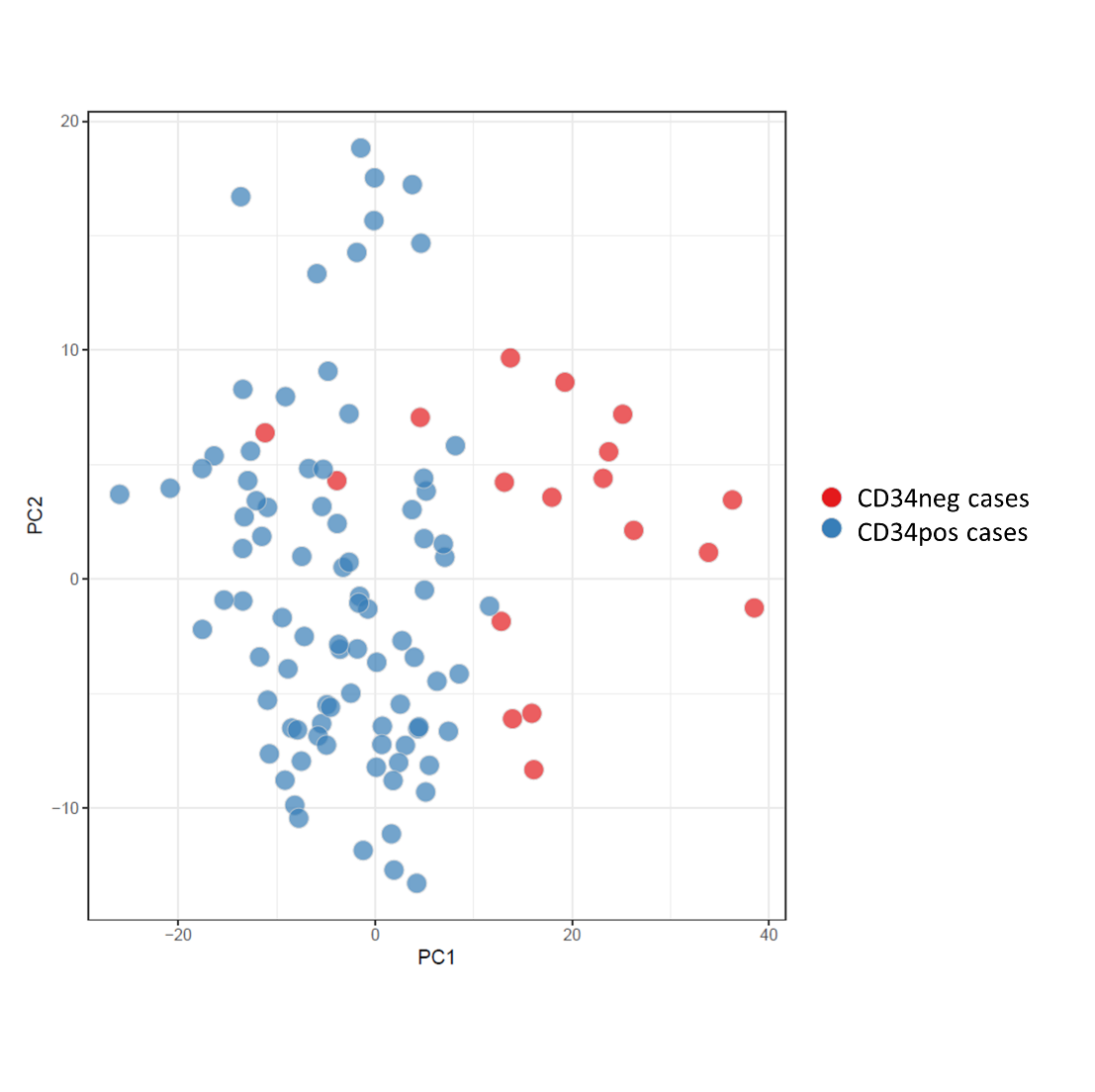


**Figure S4** **Principal component analysis of CD34 positive and negative cases**

Principal component analysis showing a moderately clear separation of the CD34 positive and CD34 negative cases (n=105)

**
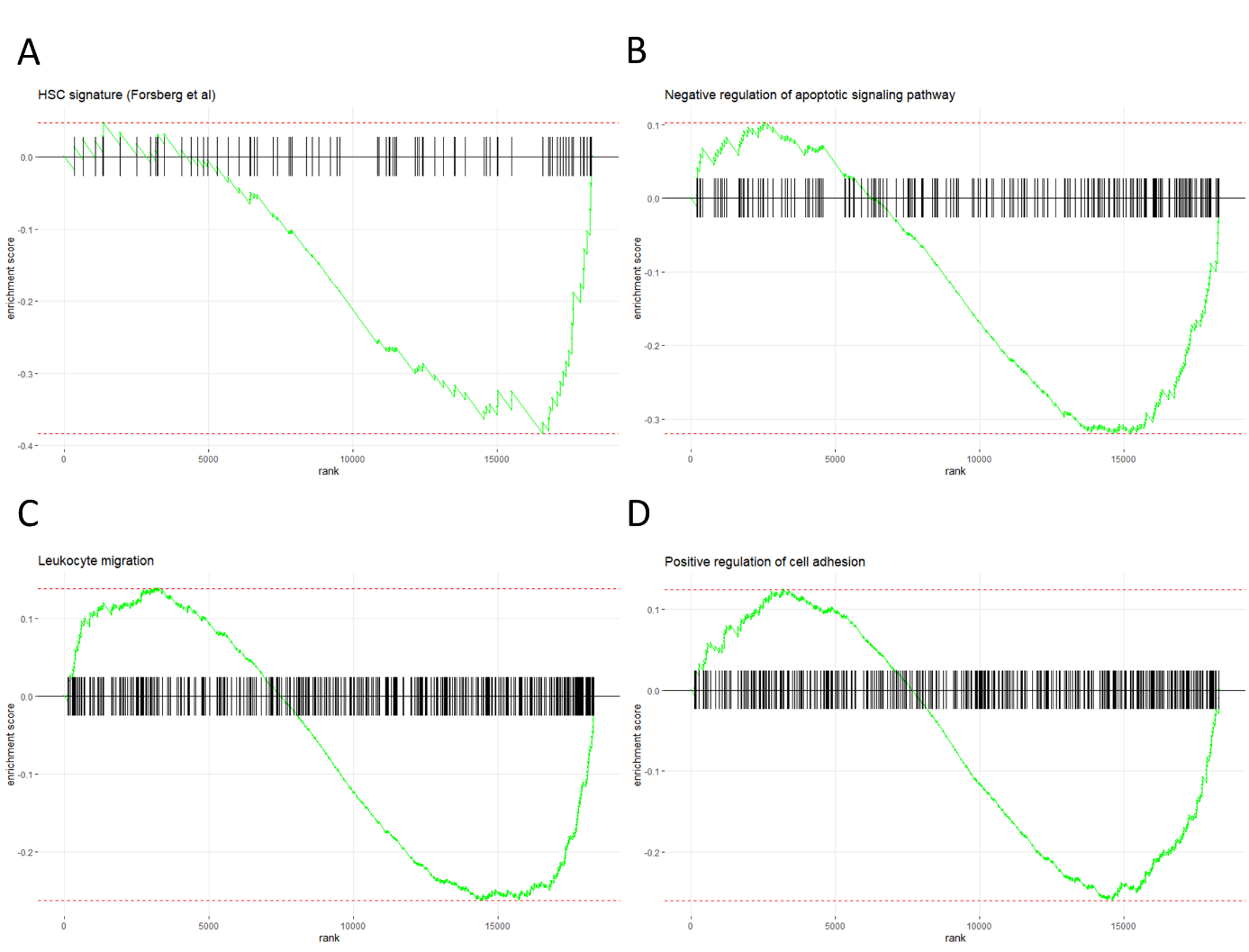
**

**Figure S5** **Gene set enrichment analysis of HSC signature genes and GO biological processes**

A: Gene set enrichment analysis (GSEA) of genes associated with healthy, quiescent hematopoietic stem cells identified by Forsberg et al[27]. Of 93 murine genes, 84 could be directly assigned to a human homologue without insecurities involving multimapping/paralogues and have been included in the analysis. A significant enrichment was seen among CD34 positive leukemias (ES: -0.38, NES: -1.87, p=0.0007). B-D: Three selected GO biological processes identified in PPI network analysis were examined with GSEA. Significant enrichment within CD34pos leukemias were found for all three: B: Negative regulation of apoptotic signaling pathway (padj=0.0017, ES: -0.32, NES: -1.65). C: Leukocyte migration (padj=0.0046, ES: -0.26, NES: -1.46) D: Positive regulation of cell adhesion (padj=0.0051, ES: -0.26, NES: -1.43).

ES: enrichment score. GO: Gene ontology. NES: enrichment score normalized to mean enrichment of random samples of the same size. Padj: FDR-corrected p-value (Benjamini-Hochberg).

**Figures S6-15** **Significantly altered PPI networks between CD34 positive and CD34 negative leukemias.**

Top panels illustrate by colour the top 3 overrepresented gene ontology biological processes listed in table S4. Bottom panels illustrate direction of gene expression. Red/arrow up: upregulated in CD34negative leukemias, blue/arrow down: upregulated in CD34positive leukemias. Significance level was set to an FDR adjusted p-value (q-value) of <0.05. The ten networks are also available as a Cytoscape (cytoscape.org) session file upon request.

**
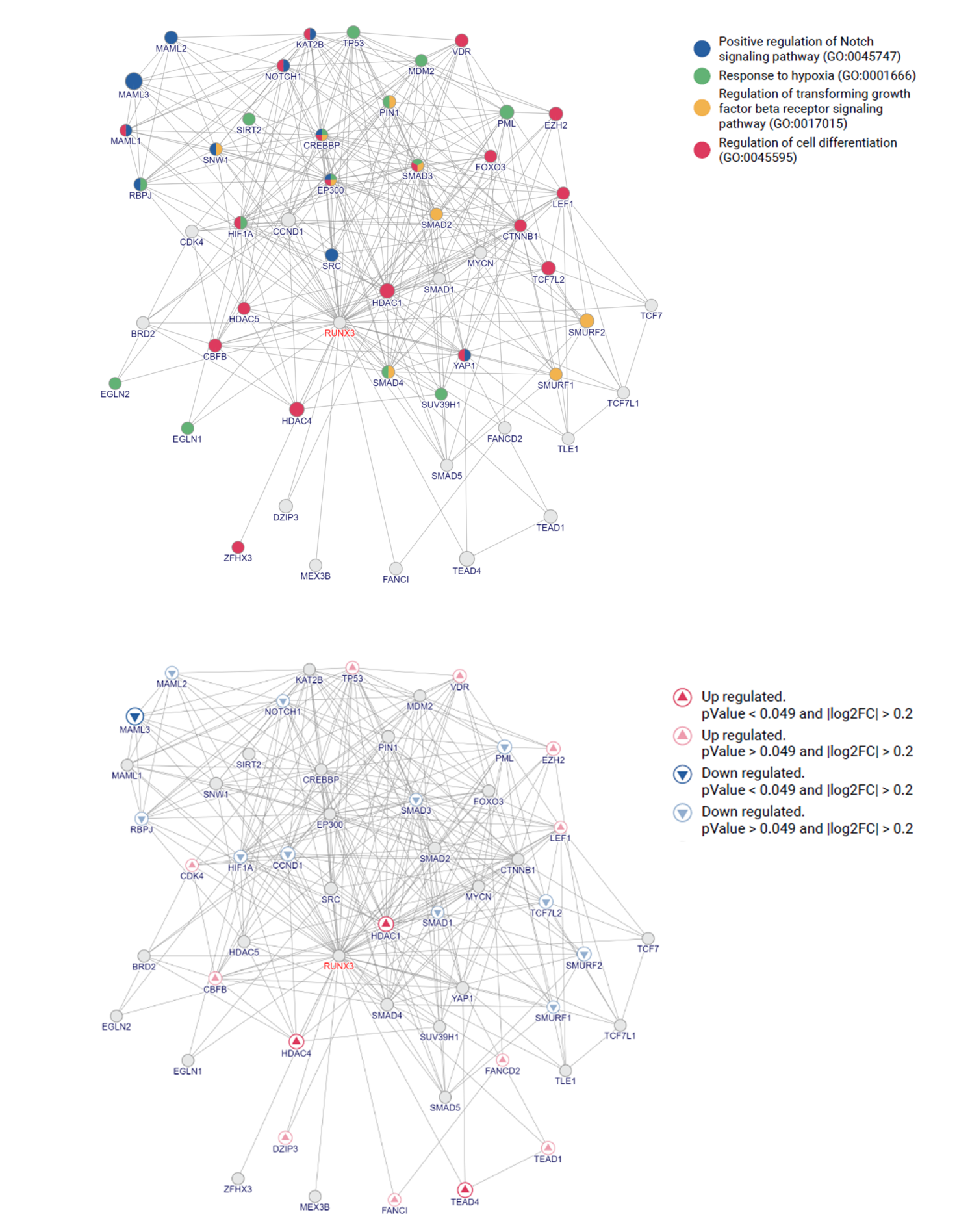
**

**S6 Network 1**

**
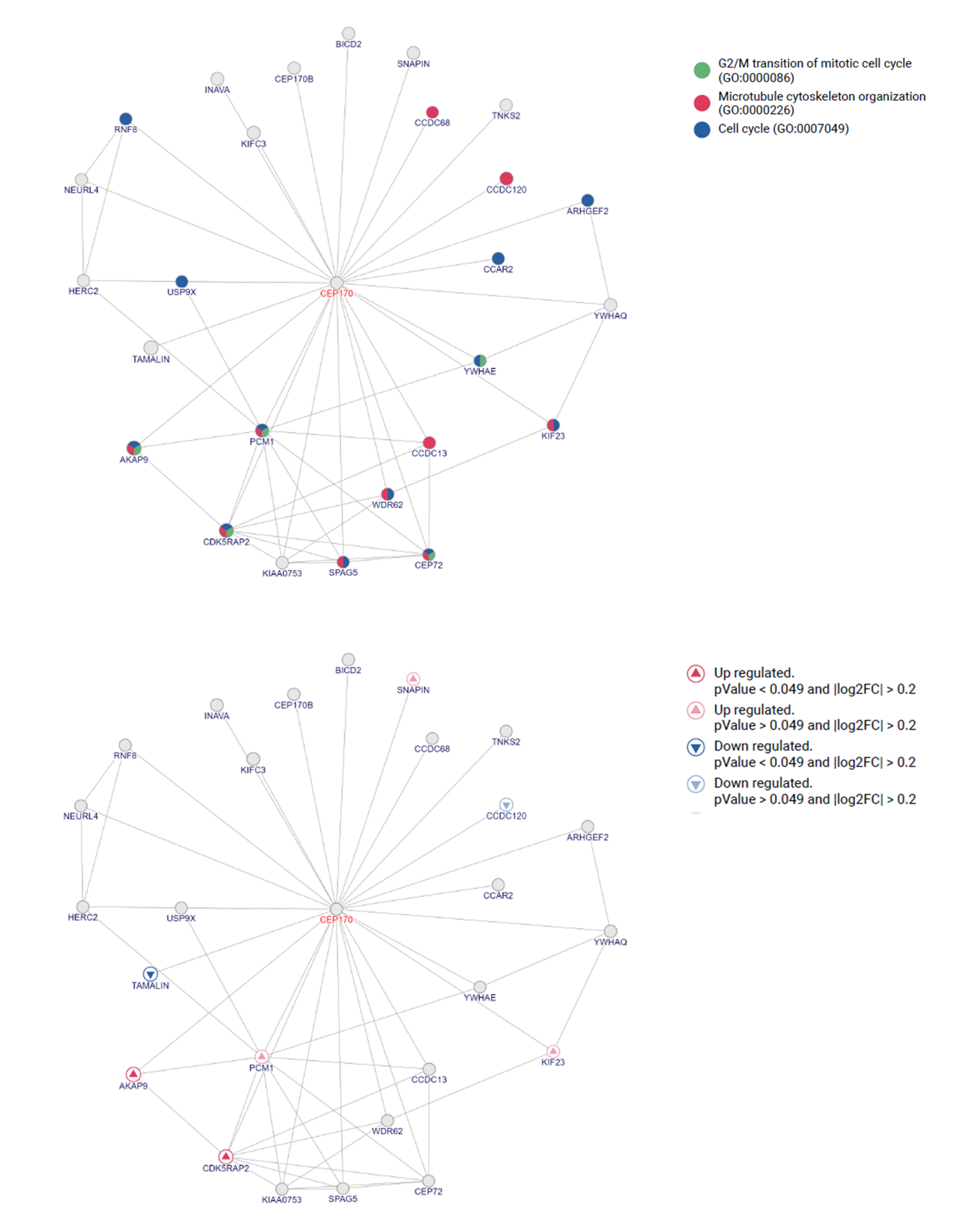
**

**S7 Network 2**

**
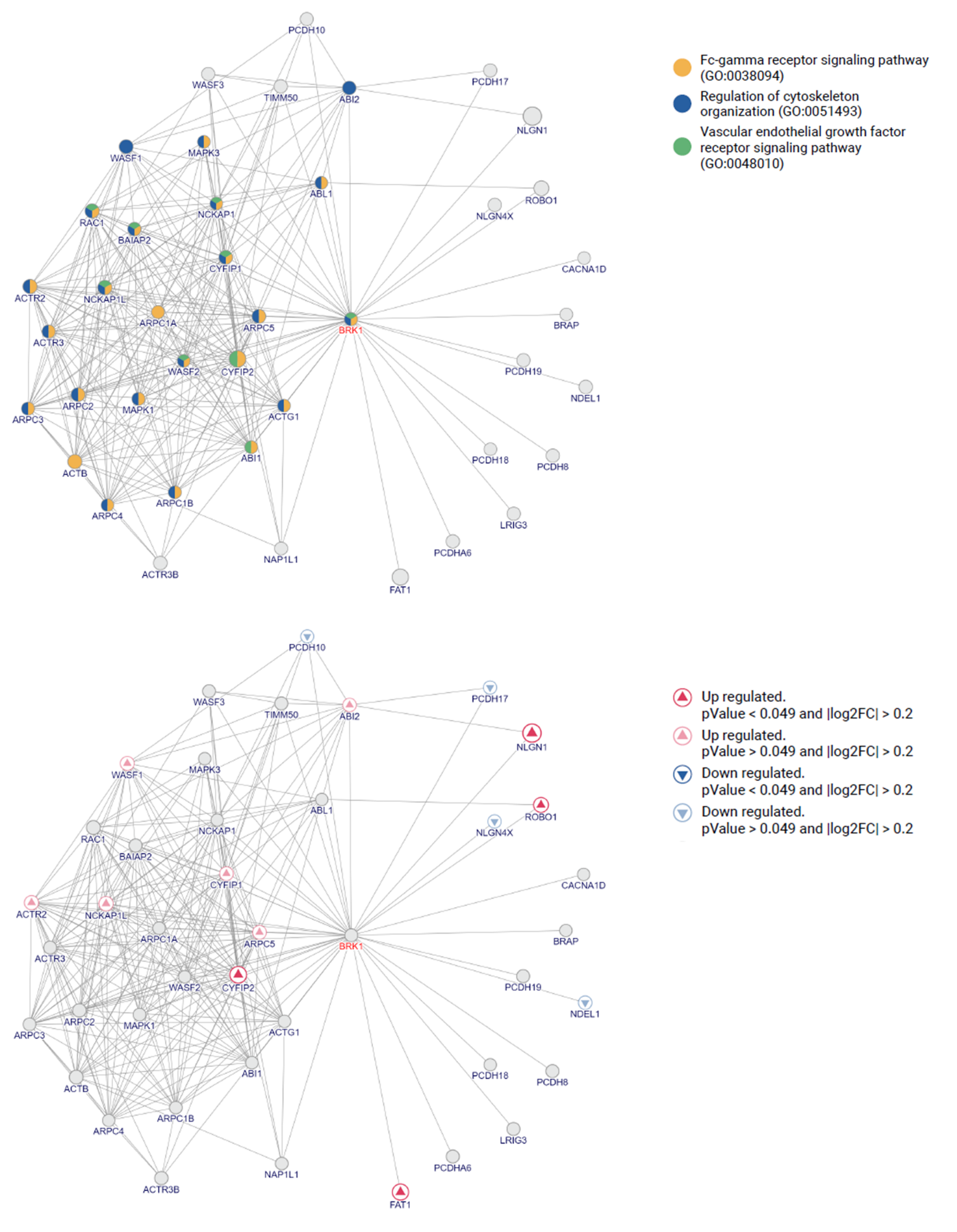
**

**S8 Network 3**

**
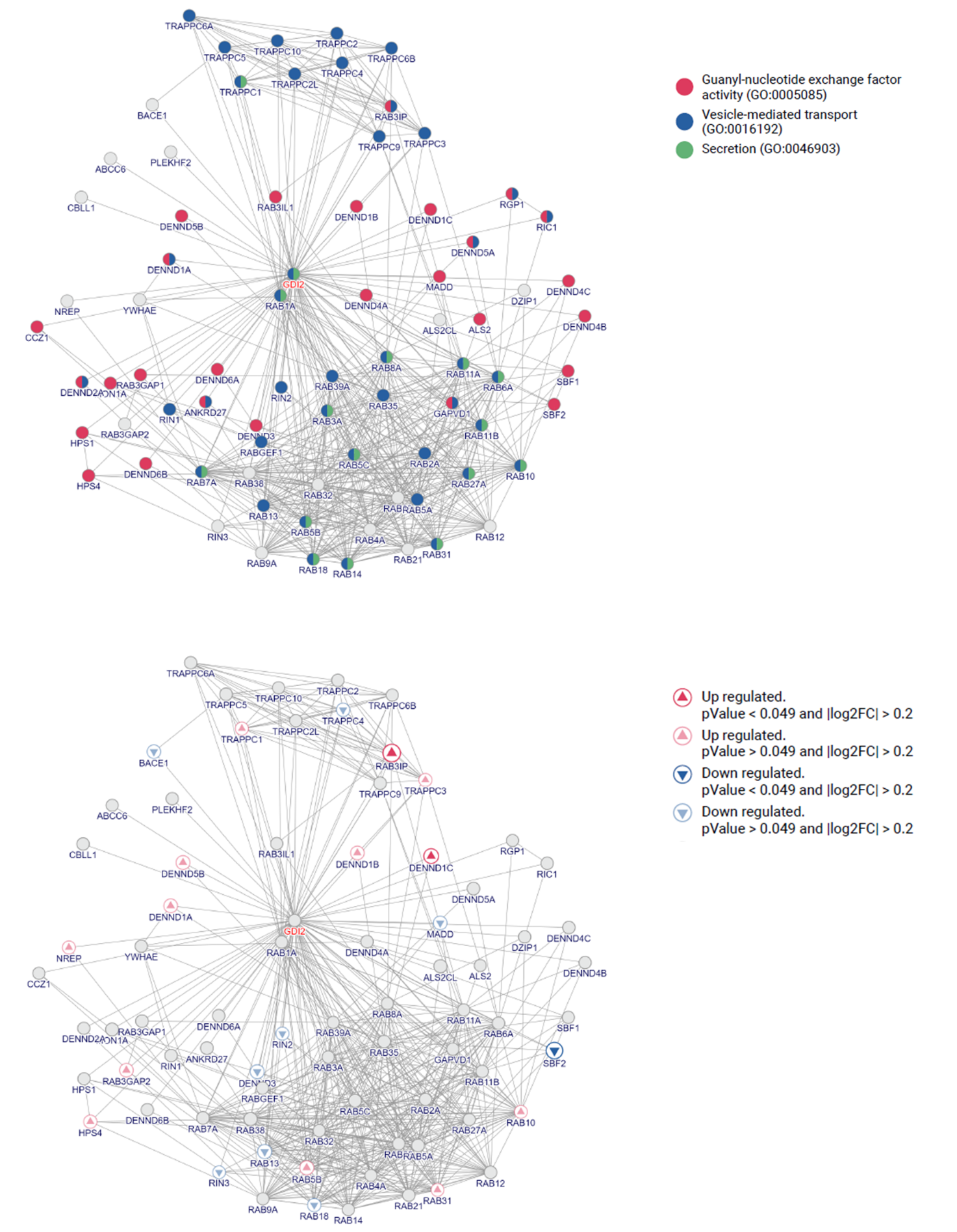
**

**S9 Network 4**

**
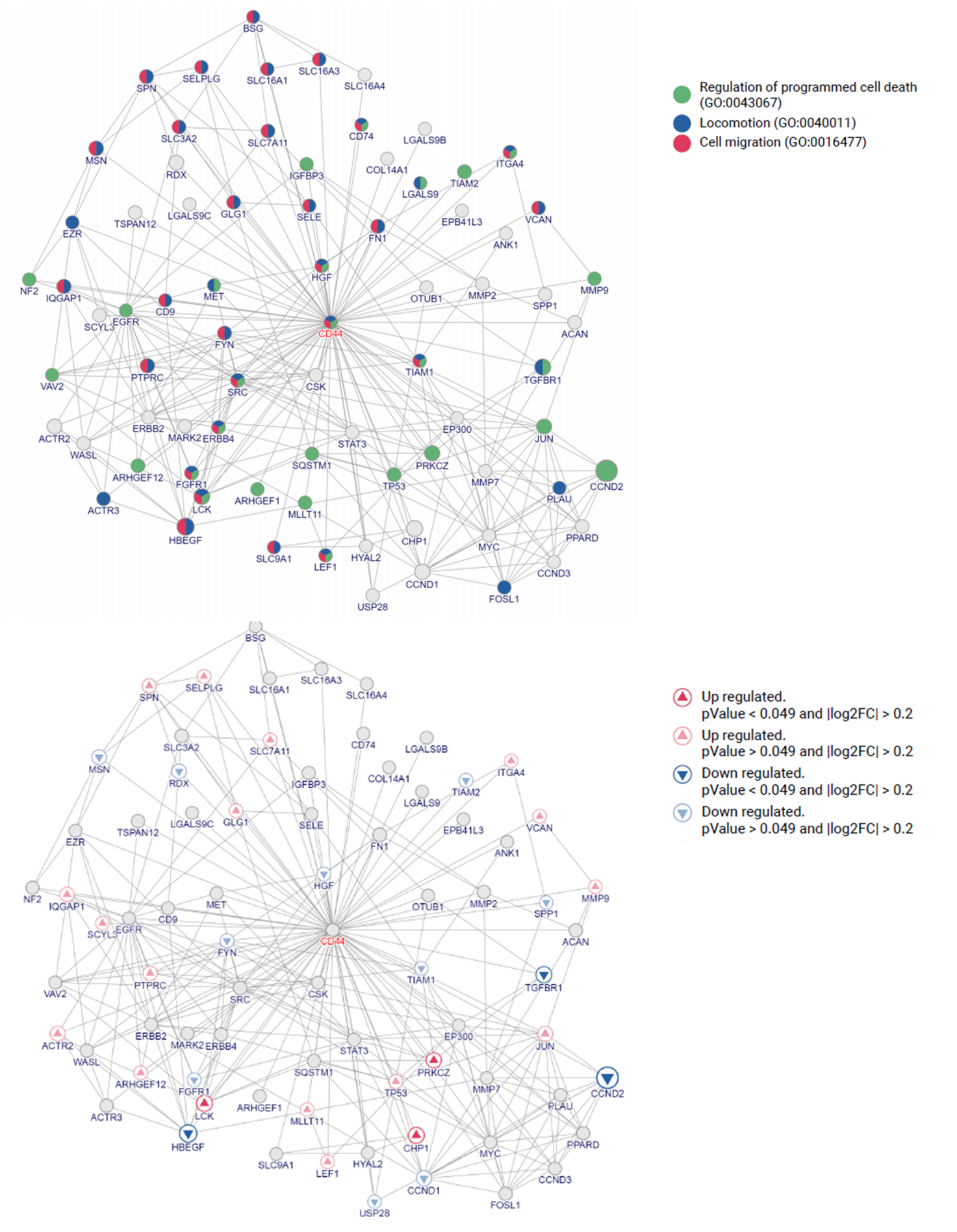
**

**S10 Network 5**

**
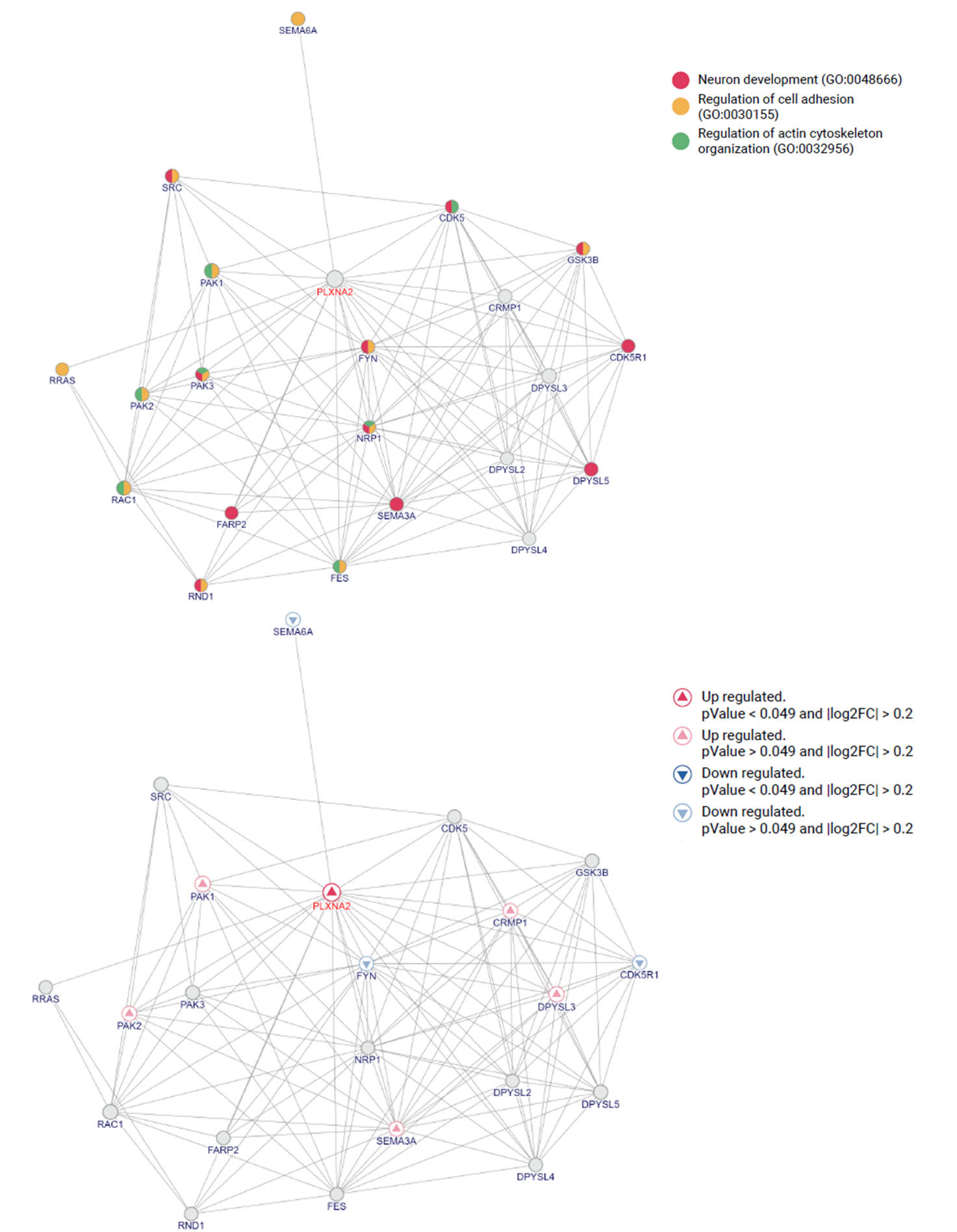
**

**S11 Network 6**

**
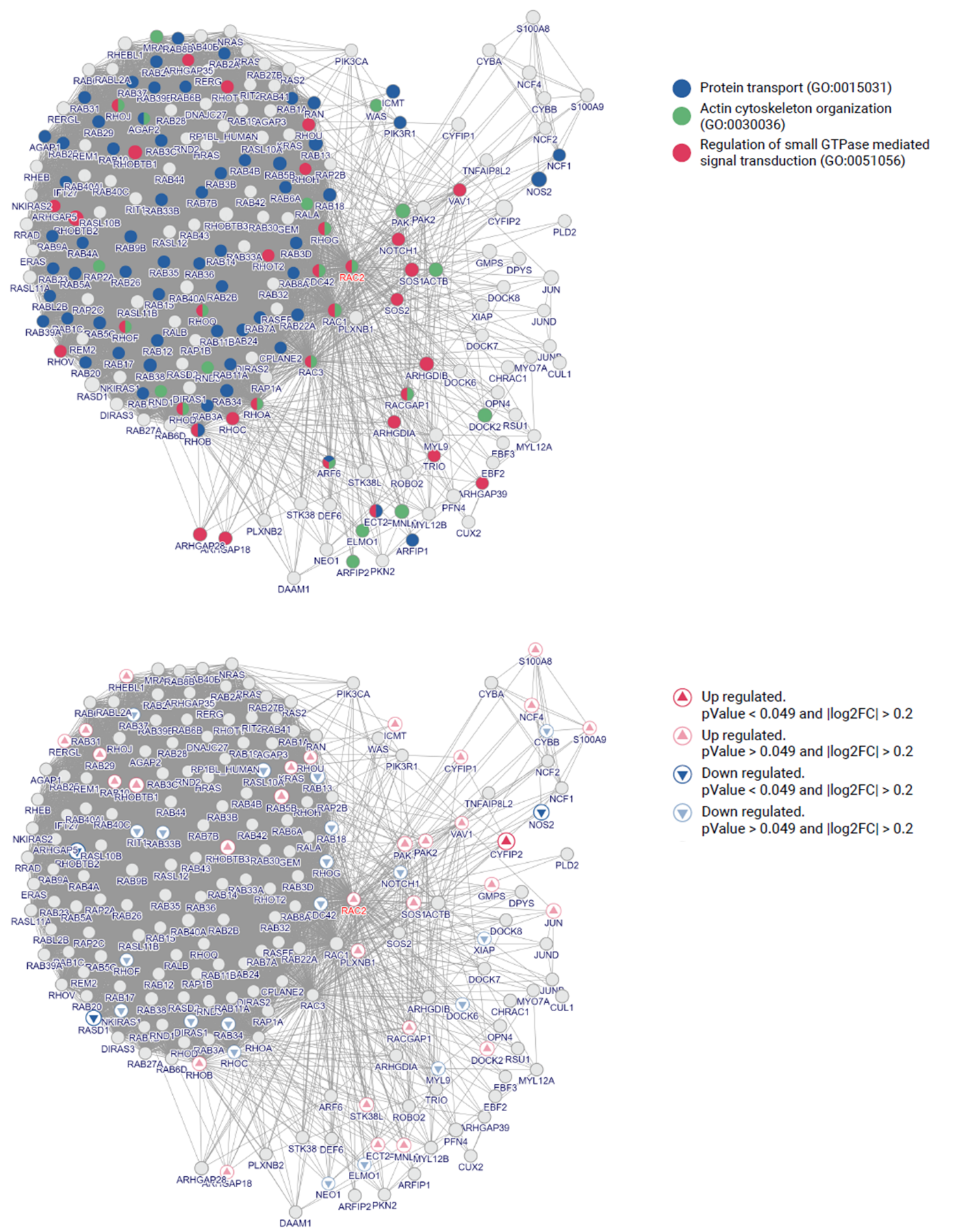
**

**S12 Network 7**

**
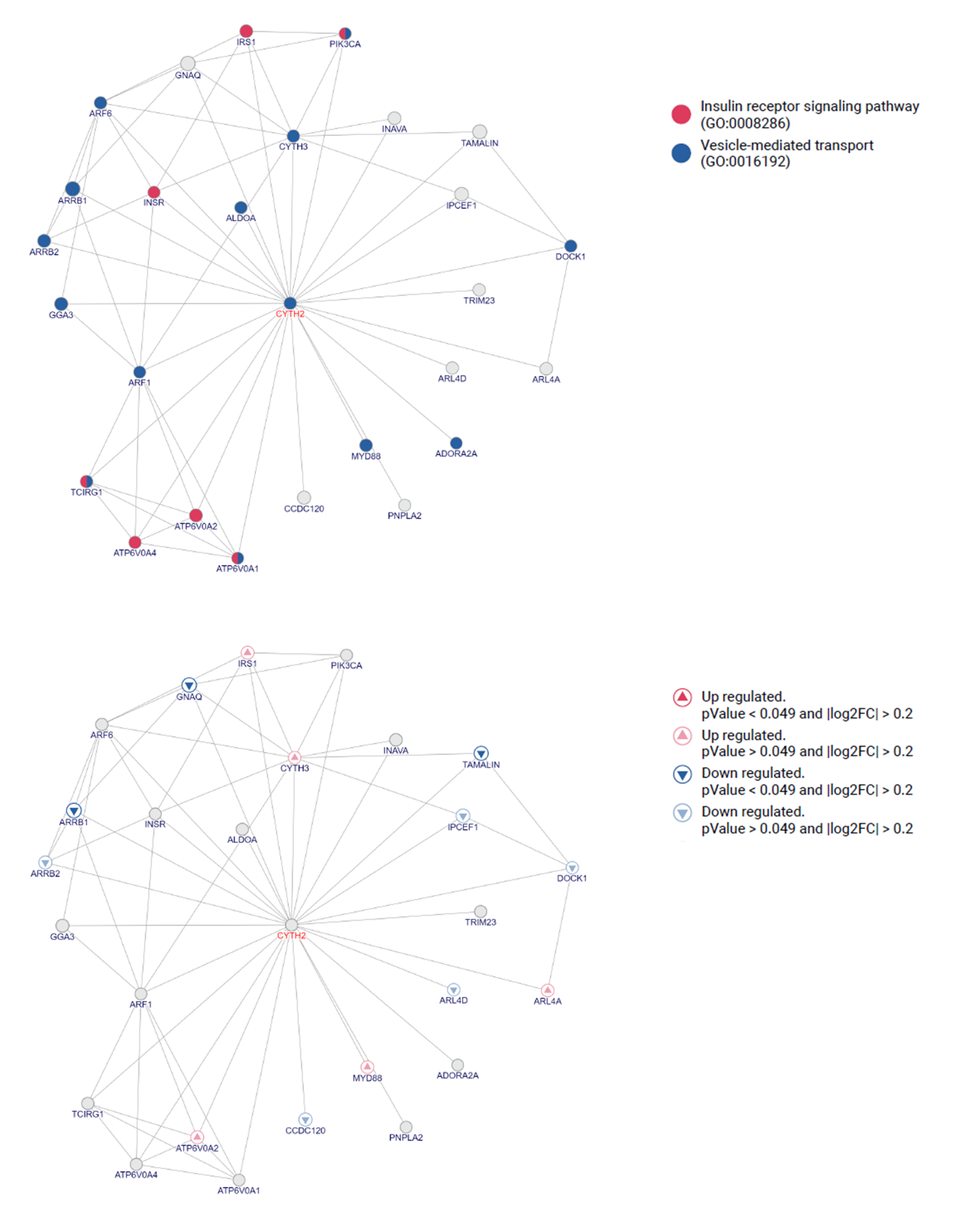
**

**S13 Network 8**

**
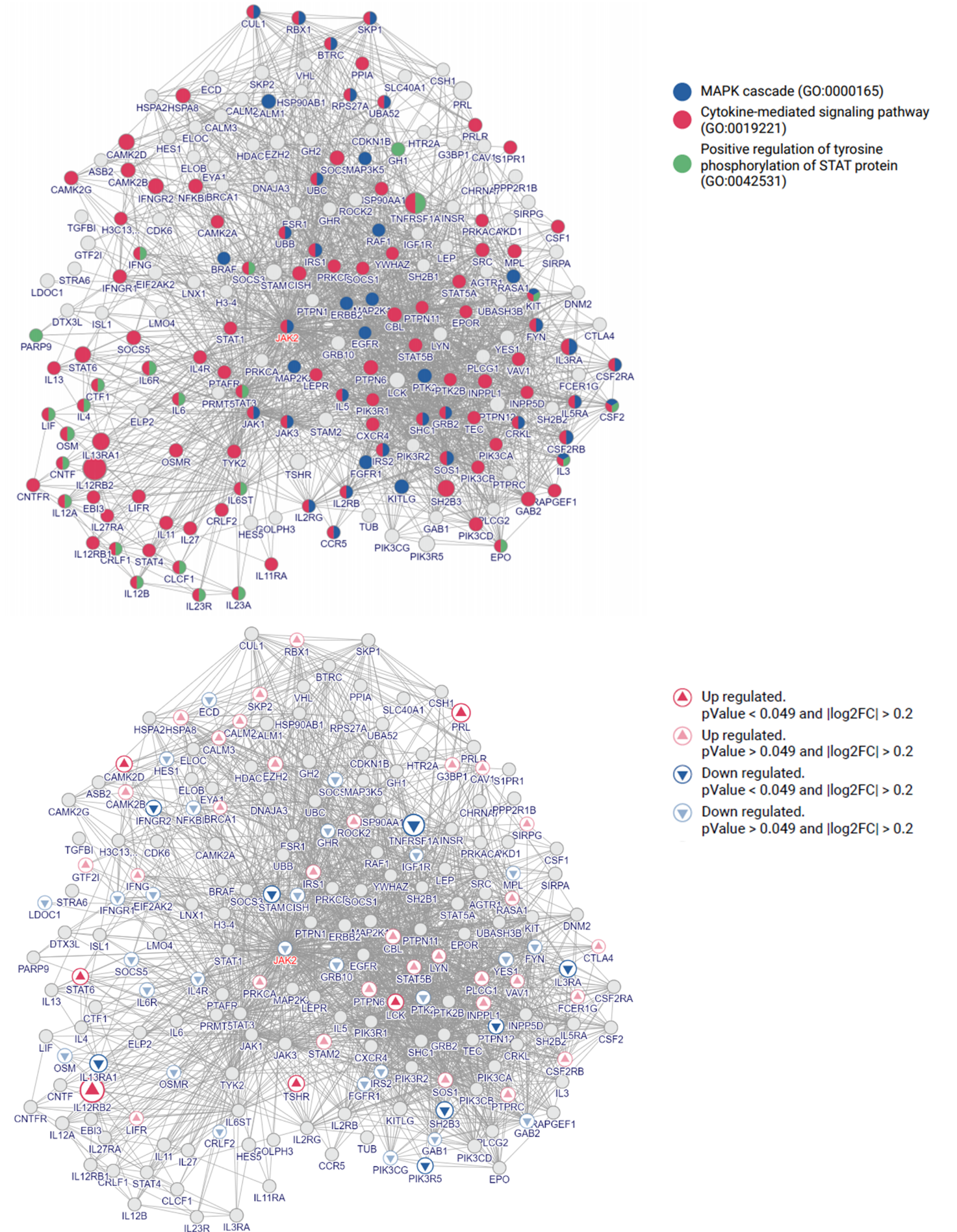
**

**S14 Network 9**

**
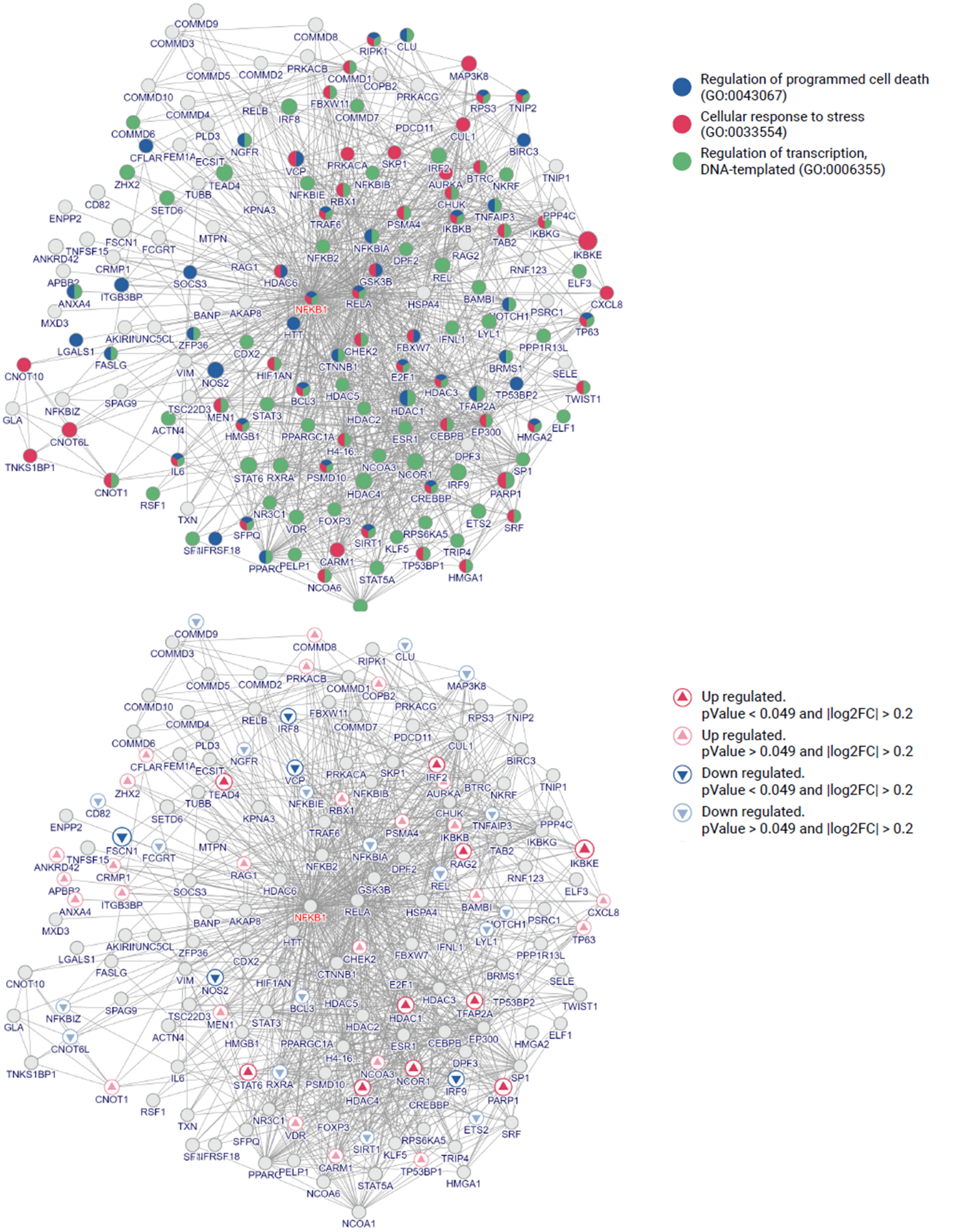
**

**S15 Network 10**


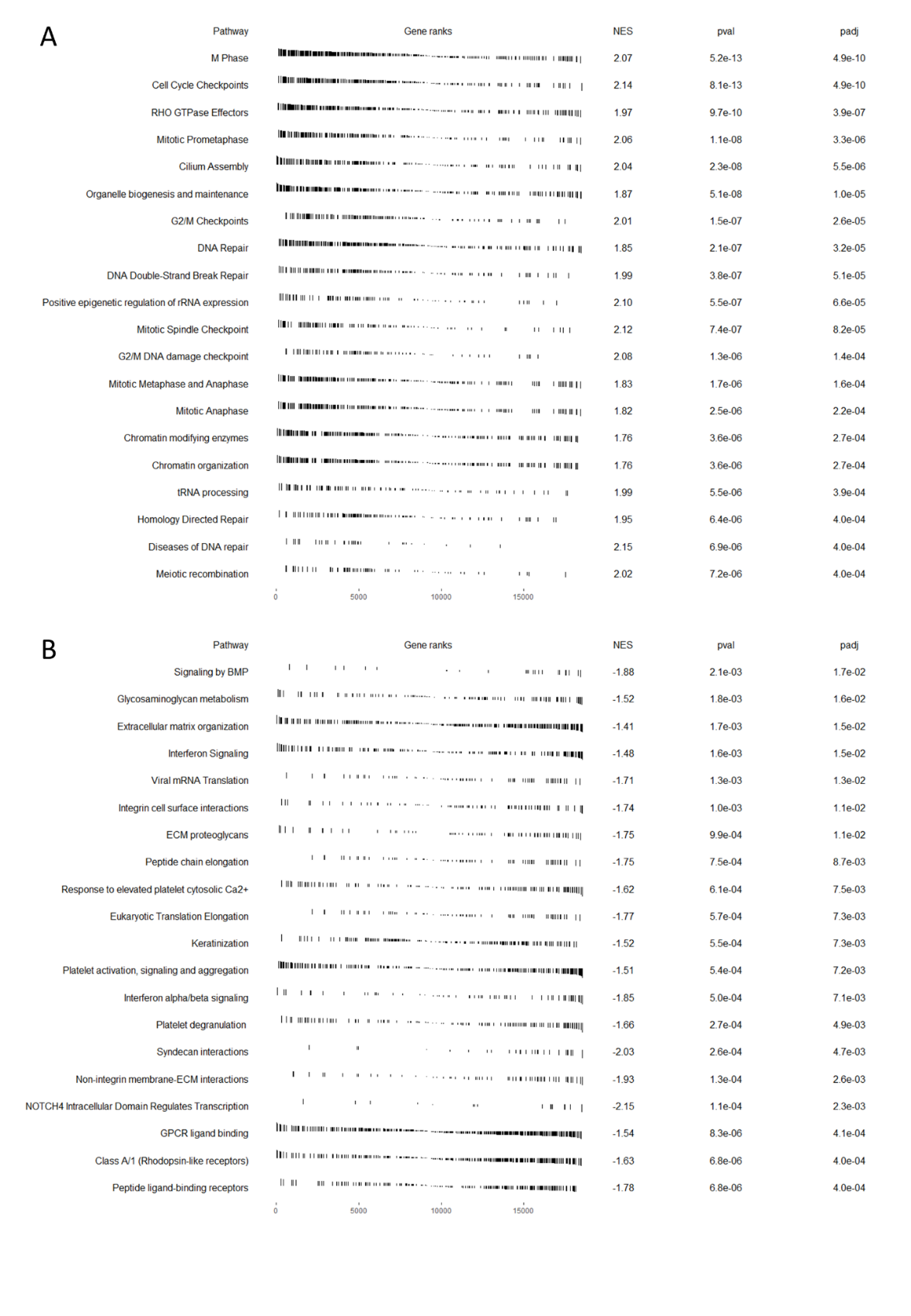


**Figure S16 Top ten enriched pathways in CD34 negative and CD34 positive leukemias**

Top ten enriched pathways in CD34neg (A) and CD34pos (B) leukemias. Gene set enrichment analysis using Reactome pathways. NES: enrichment score normalized to mean enrichment of random samples of the same size. Padj: FDR-corrected p-value (Benjamini-Hochberg).

**Supplemental references**

1. Tomiyama N, Ikeda R, Nishizawa Y, Masuda S, Tajitsu Y, Takeda Y: **S100A16 up-regulates Oct4 and Nanog expression in cancer stem-like cells of Yumoto human cervical carcinoma cells**. *Oncol Lett* 2018, **15**(6):9929-9933.

2. Balise VD, Saito-Reis CA, Gillette JM: **Tetraspanin Scaffold Proteins Function as Key Regulators of Hematopoietic Stem Cells**. *Front Cell Dev Biol* 2020, **8**:598.

3. Amirizadeh N, Oodi A, Mehrasa R, Nikougoftar M: **Apoptosis, DAP-Kinase1 Expression and the Influences of Cytokine Milieu and Mesenchymal Stromal Cells on Ex Vivo Expansion of Umbilical Cord Blood-Derived Hematopoietic Stem Cells**. *Indian J Hematol Blood Transfus* 2016, **32**(1):67-77.

4. Fortunel NO, Otu HH, Ng HH, Chen J, Mu X, Chevassut T, Li X, Joseph M, Bailey C, Hatzfeld JA *et al*: **Comment on " 'Stemness': transcriptional profiling of embryonic and adult stem cells" and "a stem cell molecular signature"**. *Science* 2003, **302**(5644):393; author reply 393.

5. Emori M, Tsukahara T, Murase M, Kano M, Murata K, Takahashi A, Kubo T, Asanuma H, Yasuda K, Kochin V *et al*: **High expression of CD109 antigen regulates the phenotype of cancer stem-like cells/cancer-initiating cells in the novel epithelioid sarcoma cell line ESX and is related to poor prognosis of soft tissue sarcoma**. *PLoS One* 2013, **8**(12):e84187.

6. Huang WC, Tung SL, Chen YL, Chen PM, Chu PY: **IFI44L is a novel tumor suppressor in human hepatocellular carcinoma affecting cancer stemness, metastasis, and drug resistance via regulating met/Src signaling pathway**. *BMC Cancer* 2018, **18**(1):609.

7. Shin Y, Won Y, Yang JI, Chun JS: **CYTL1 regulates bone homeostasis in mice by modulating osteogenesis of mesenchymal stem cells and osteoclastogenesis of bone marrow-derived macrophages**. *Cell Death Dis* 2019, **10**(2):47.

8. Kozar K, Ciemerych MA, Rebel VI, Shigematsu H, Zagozdzon A, Sicinska E, Geng Y, Yu Q, Bhattacharya S, Bronson RT *et al*: **Mouse development and cell proliferation in the absence of D-cyclins**. *Cell* 2004, **118**(4):477-491.

9. Eto H, Ishimine H, Kinoshita K, Watanabe-Susaki K, Kato H, Doi K, Kuno S, Kurisaki A, Yoshimura K: **Characterization of human adipose tissue-resident hematopoietic cell populations reveals a novel macrophage subpopulation with CD34 expression and mesenchymal multipotency**. *Stem Cells Dev* 2013, **22**(6):985-997.

10. Zhu P, Zhu X, Wu J, He L, Lu T, Wang Y, Liu B, Ye B, Sun L, Fan D *et al*: **IL-13 secreted by ILC2s promotes the self-renewal of intestinal stem cells through circular RNA circPan3**. *Nat Immunol* 2019, **20**(2):183-194.

11. Krampera M, Pasini A, Rigo A, Scupoli MT, Tecchio C, Malpeli G, Scarpa A, Dazzi F, Pizzolo G, Vinante F: **HB-EGF/HER-1 signaling in bone marrow mesenchymal stem cells: inducing cell expansion and reversibly preventing multilineage differentiation**. *Blood* 2005, **106**(1):59-66.

12. Nguyen TM, Arthur A, Zannettino AC, Gronthos S: **EphA5 and EphA7 forward signaling enhances human hematopoietic stem and progenitor cell maintenance, migration, and adhesion via Rac1 activation**. *Exp Hematol* 2017, **48**:72-78.

13. Coste C, Neirinckx V, Sharma A, Agirman G, Rogister B, Foguenne J, Lallemend F, Gothot A, Wislet S: **Human bone marrow harbors cells with neural crest-associated characteristics like human adipose and dermis tissues**. *PLoS One* 2017, **12**(7):e0177962.

14. Jafari A, Qanie D, Andersen TL, Zhang Y, Chen L, Postert B, Parsons S, Ditzel N, Khosla S, Johansen HT *et al*: **Legumain Regulates Differentiation Fate of Human Bone Marrow Stromal Cells and Is Altered in Postmenopausal Osteoporosis**. *Stem Cell Reports* 2017, **8**(2):373-386.

15. Arthur A, Nguyen TM, Paton S, Zannettino ACW, Gronthos S: **Loss of EfnB1 in the osteogenic lineage compromises their capacity to support hematopoietic stem/progenitor cell maintenance**. *Exp Hematol* 2019, **69**:43-53.

16. Horvay K, Jarde T, Casagranda F, Perreau VM, Haigh K, Nefzger CM, Akhtar R, Gridley T, Berx G, Haigh JJ *et al*: **Snai1 regulates cell lineage allocation and stem cell maintenance in the mouse intestinal epithelium**. *EMBO J* 2015, **34**(10):1319-1335.

17. Wang YH, Lin CC, Yao CY, Hsu CL, Hou HA, Tsai CH, Chou WC, Tien HF: **A 4-gene leukemic stem cell score can independently predict the prognosis of myelodysplastic syndrome patients**. *Blood Adv* 2020, **4**(4):644-654.

18. Monlish DA, Greenberg ZJ, Bhatt ST, Leonard KM, Romine MP, Dong Q, Bendesky L, Duncavage EJ, Magee JA, Schuettpelz LG: **TLR2/6 signaling promotes the expansion of premalignant hematopoietic stem and progenitor cells in the NUP98-HOXD13 mouse model of MDS**. *Exp Hematol* 2020, **88**:42-55.

19. Wang WT, Chen TQ, Zeng ZC, Pan Q, Huang W, Han C, Fang K, Sun LY, Yang QQ, Wang D *et al*: **The lncRNA LAMP5-AS1 drives leukemia cell stemness by directly modulating DOT1L methyltransferase activity in MLL leukemia**. *J Hematol Oncol* 2020, **13**(1):78.

20. Panchal H, Wansbury O, Parry S, Ashworth A, Howard B: **Neuregulin3 alters cell fate in the epidermis and mammary gland**. *BMC Dev Biol* 2007, **7**:105.

21. Marchand M, Horcajadas JA, Esteban FJ, McElroy SL, Fisher SJ, Giudice LC: **Transcriptomic signature of trophoblast differentiation in a human embryonic stem cell model**. *Biol Reprod* 2011, **84**(6):1258-1271.

22. Pastushenko I, Mauri F, Song Y, de Cock F, Meeusen B, Swedlund B, Impens F, Van Haver D, Opitz M, Thery M *et al*: **Fat1 deletion promotes hybrid EMT state, tumour stemness and metastasis**. *Nature* 2021, **589**(7842):448-455.

23. Yang Z, Li C, Fan Z, Liu H, Zhang X, Cai Z, Xu L, Luo J, Huang Y, He L *et al*: **Single-cell Sequencing Reveals Variants in ARID1A, GPRC5A and MLL2 Driving Self-renewal of Human Bladder Cancer Stem Cells**. *Eur Urol* 2017, **71**(1):8-12.

24. Niu X, Li J, Zhao X, Wang Q, Wang G, Hou R, Li X, An P, Yin G, Zhang K: **Dermal mesenchymal stem cells: a resource of migration-associated function in psoriasis?** *Stem Cell Res Ther* 2019, **10**(1):54.

25. Park SM, Cho H, Thornton AM, Barlowe TS, Chou T, Chhangawala S, Fairchild L, Taggart J, Chow A, Schurer A *et al*: **IKZF2 Drives Leukemia Stem Cell Self-Renewal and Inhibits Myeloid Differentiation**. *Cell Stem Cell* 2019, **24**(1):153-165 e157.

26. Caras IW: **Two cancer stem cell-targeted therapies in clinical trials as viewed from the standpoint of the cancer stem cell model**. *Stem Cells Transl Med* 2020, **9**(8):821-826.

27. Forsberg EC, Passegue E, Prohaska SS, Wagers AJ, Koeva M, Stuart JM, Weissman IL: **Molecular signatures of quiescent, mobilized and leukemia-initiating hematopoietic stem cells**. *PLoS One* 2010, **5**(1):e8785.
